# A rationally identified panel of microRNAs targets multiple oncogenic pathways to enhance chemotherapeutic effects in glioblastoma models

**DOI:** 10.1038/s41598-022-16219-x

**Published:** 2022-07-14

**Authors:** Negar Sadeghipour, Sukumar Uday Kumar, Tarik F. Massoud, Ramasamy Paulmurugan

**Affiliations:** 1 Molecular Imaging Program at Stanford (MIPS), Stanford University School of Medicine, Stanford, CA USA; 2 Cellular Pathway Imaging Laboratory (CPIL), The Canary Center at Stanford for Cancer Early Detection, Stanford University School of Medicine, Palo Alto, CA USA; 3 Cellular Pathway Imaging Laboratory (CPIL), Molecular Imaging Program at Stanford (MIPS), Canary Center for Cancer Early Detection at Stanford, Stanford University School of Medicine, 3155 Porter Drive, Palo Alto, CA 94304 USA; 4 Laboratory of Experimental and Molecular Neuroimaging (LEMNI), Molecular Imaging Program at Stanford (MIPS), Stanford University School of Medicine, 3155 Porter Drive, Palo Alto, CA 94304 USA

**Keywords:** Cancer therapy, Tumour heterogeneity

## Abstract

Glioblastoma (GBM) is the most common malignant brain tumor. Available treatments have limited success because most patients develop chemoresistance. Alternative strategies are required to improve anticancer effects of current chemotherapeutics while limiting resistance. Successful targeting of microRNAs (miRNAs) as regulators of gene expression can help reprogram GBM cells to better respond to chemotherapy. We aimed to identify a panel of miRNAs that target multiple oncogenic pathways to improve GBM therapy. We first identified differentially expressed miRNAs and tested if their target genes play central roles in GBM signaling pathways by analyzing data in the Gene Expression Omnibus and The Cancer Genome Atlas databases. We then studied the effects of different combinations of these miRNAs in GBM cells by delivering synthetic miRNAs using clinically compatible PLGA-PEG nanoparticles prior to treatment with temozolomide (TMZ) or doxorubicin (DOX). The successful miRNA panel was tested in mice bearing U87-MG cells co-treated with TMZ. We identified a panel of five miRNAs (miRNA-138, miRNA-139, miRNA-218, miRNA-490, and miRNA-21) and their oncogenic targets (*CDK6, ZEB1, STAT3, TGIF2,* and *SMAD7*) that cover four different signaling pathways (cell proliferation and apoptotic signaling, invasion and metastasis, cytokine signaling, and stemness) in GBM. We observed significant in vitro and in vivo enhancement of therapeutic efficiency of TMZ and DOX in GBM models. The proposed combination therapy using rationally selected miRNAs and chemotherapeutic drugs is effective owing to the ability of this specific miRNA panel to better target multiple genes associated with the hallmarks of cancer.

## Introduction

Glioblastoma (GBM) is the most common and aggressive primary brain tumor in adults. Clinical chemotherapeutic drugs, including temozolomide (TMZ), can extend the overall survival of GBM patients by about two months on average^1,2^. However, patients show resistance to these drugs owing to a multitude of factors, including intratumoral heterogeneities^3,4^, the presence of cancer stem cells^5^, activation of multidrug resistance, and epithelial–mesenchymal transition (E-MT)^6^. Moreover, alterations in several other oncogenic signaling mechanisms such as overexpression of EGFR^7^ and mutations in p53 tumor suppressor gene^8^ lead to tumor recurrence and metastasis. In addition, effective drug delivery to GBM remains a challenge, mainly owing to the blood–brain barrier (BBB) and blood-tumor barrier, as well as rapid clearance of therapeutics from the brain^9–11^. Dose-limiting toxicity also diminishes the amount of drugs that can be delivered for effective treatment. These challenges could be partly addressed if nontoxic sensitization of cancer cells were possible prior to exposure to chemotherapy.

MicroRNAs (miRNAs) are small non-coding RNA molecules that play a crucial role in gene regulation. Certain miRNAs are differentially expressed (i.e., significantly up- or downregulated) in cancer compared to normal tissues, and they could act as cancer suppressive miRNAs or oncomiRs^12,13^. Studies have found that some of these miRNAs can enhance the therapeutic effects of traditional drugs^14^ and enhance GBM chemosensitivity to TMZ^15–17^. However, it has been challenging for many reasons to conceive and adopt new therapeutic miRNA strategies^18,19^. Finding the right combination of miRNAs among the myriads of potential candidates to target and then achieve an effective anticancer effect can be a daunting proposition. It would be more useful to initially develop a comprehensive method to select relevant miRNAs, along with a rigorous target gene identification method to increase the likelihood of success in achieving multiple and simultaneous anticancer effects of miRNAs for improved GBM treatment.

Here, we identified a panel of miRNAs with well-known target genes involved in one of the following four signaling pathways for further experimental validation in in vitro and in vivo studies: (1) apoptotic and growth signaling, (2) invasion and metastasis, (3) cytokine signaling, and (4) stemness. We leveraged bioinformatics tools to analyze GBM and normal brain tissue data from available databases, such as The Cancer Genomic Atlas (TCGA) and Gene Expression Omnibus (GEO), to establish different criteria that help narrow down our search for relevant differentially expressed miRNAs and their target gene identifications. We tested different combinations of identified miRNA mimics and/or antisense miRNAs, in the presence and absence of TMZ or doxorubicin (DOX) in GBM cells of different phenotypes in culture using miRNA-loaded PLGA-PEG nanoparticles, as reported previously^20,21^. Based on the in vitro results, we selected one combination and evaluated that in a GBM mouse model.

## Results

### In silico analysis

#### Identification of dysregulated miRNAs as therapeutic targets in GBM

**Figure 1:**
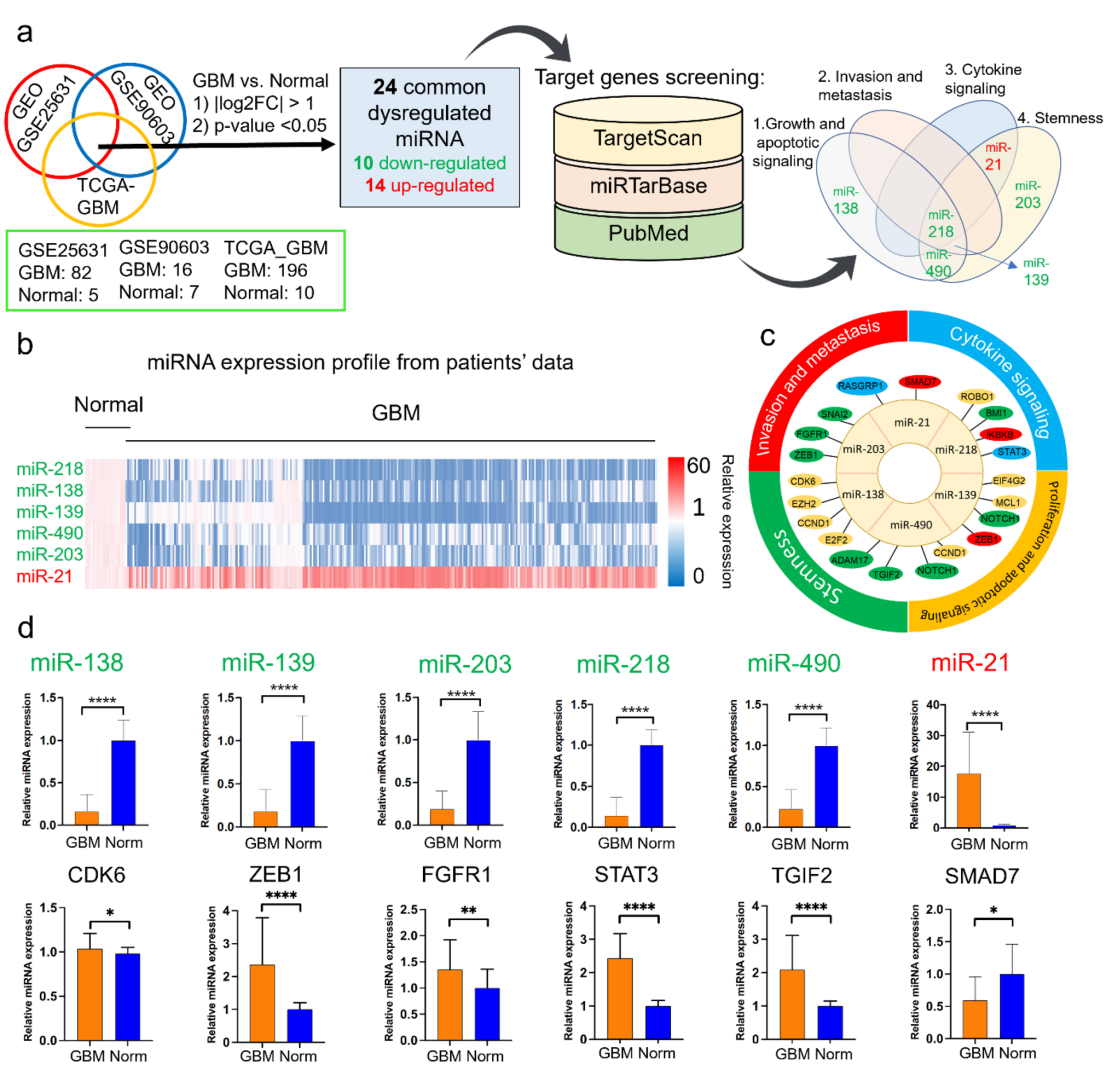
Differentially expressed miRNAs selected for applications in GBM treatment and their target genes expression. (**a**) Two datasets from GEO and one from TCGA were selected for this study. Commonly dysregulated miRNAs were selected by applying two selection criteria, and the results identified 24 differentially expressed miRNAs in GBM. Target genes for each miRNA were identified. Gene expression in GBM vs. normal brain tissue, survival analysis and correlation between the expression of miRNAs and their target genes were investigated for each target gene. The candidate miRNAs were distributed in four therapeutic pathways based on the identified target genes (**b**) Heatmap of the selected miRNA expressions in patient GBM tissues compared to normal. Values are normalized to the average expressions of normal brain tissue for each miRNA. Heatmap colors represent relative miRNA expression as shown in the color key. (**c**) The target genes of identified miRNAs selected for this study. Genes are color coded based on the therapeutic pathways. (**d**) Final six selected miRNAs and their representative target genes expressions in GBM and normal tissues. The target genes expressions are shown from GEPIA database. Significance levels are shown in the right margins: **adjusted *p* < 0.01; ****adjusted *p* < 0.0001. Genes and miRNAs (except SMAD7) were significant at |log2FC|> 1.

We followed the workflow shown in Fig. 1a to identify the dysregulated miRNAs associated with GBM. From the list of more than 500 miRNAs, we identified 24 miRNAs that were dysregulated in all three datasets. The complete list of the dysregulated miRNAs with their fold-change compared to normal brain tissues can be found in Supplementary Table S1.

#### Identification of target genes of dysregulated miRNAs

Fifteen out of 24 miRNAs were associated with regulating high profile oncogenic targets of therapeutic pathways in GBM (Supplementary Table S2). From the list of miRNAs that were associated with target genes, six of them had overlapping functional regulation in one or more of the selected pathways to use as a panel of miRNAs for therapeutic application: miRNA-203, miRNA-139, miRNA-490, miRNA-138, miRNA-218, and miRNA-21 (Supplementary Table S3). Figure 1b shows heat maps of the relative expression (GBM versus normal) of the six candidate miRNAs. Similarly, Fig. 1c shows the identified target genes for each miRNA, while Fig. 1d shows the expression of each miRNA and their most significant target gene in GBM versus normal brain tissue. In the list of downregulated miRNAs, *ZEB1*, *CDK6*, *FGFR1*, *STAT3*, and *TGIF2* are the highlighted target genes. On the other hand, *SMAD7* and *RASGRP1* were identified for upregulated miRNA-21.

**Figure 2:**
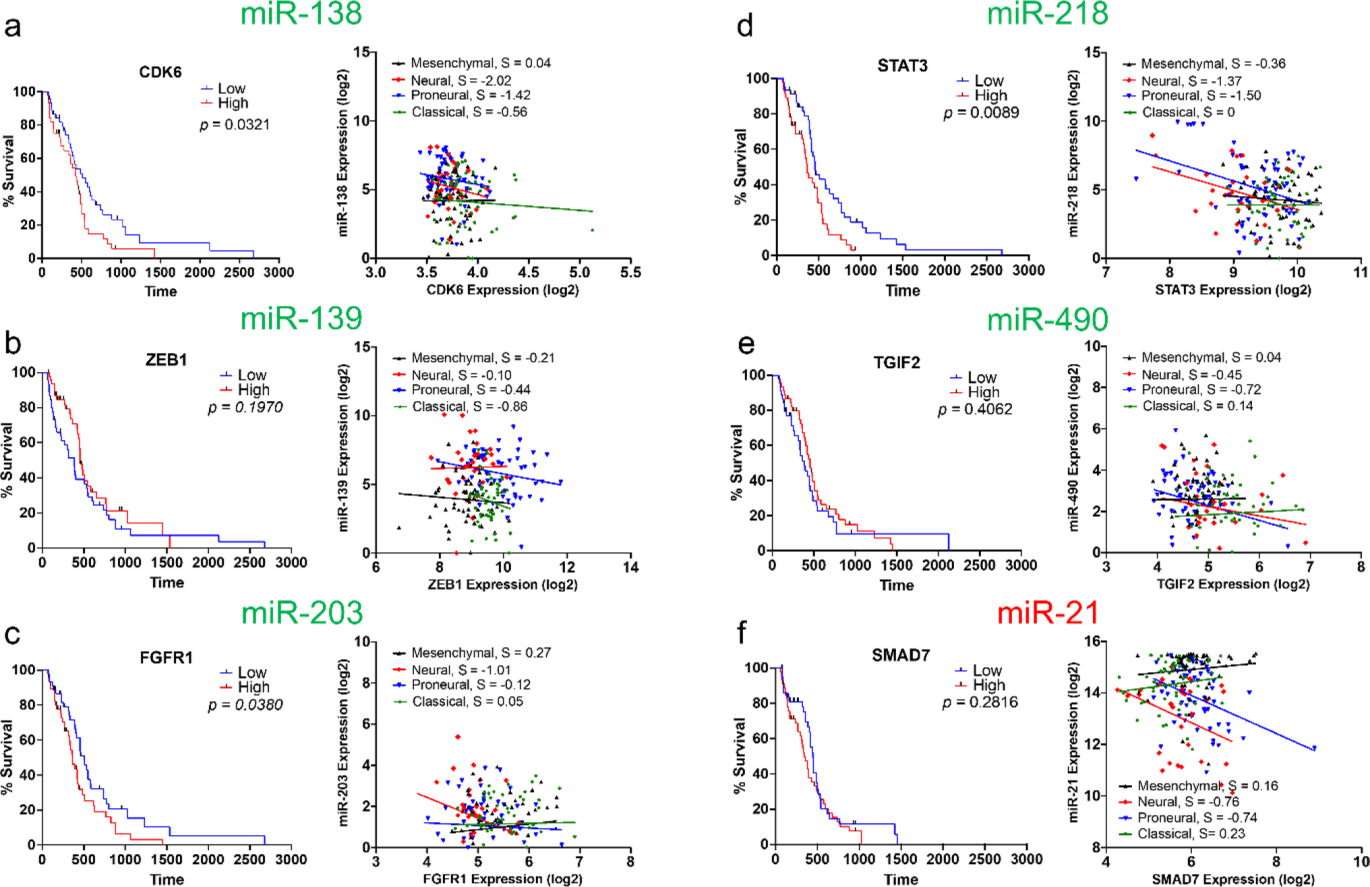
Survival curves of the target genes for each of the six selected miRNAs, and correlation of each target genes with respective miRNA expression. Correlation between the target gene and miRNA were studied in four different GBM subtypes: mesenchymal, neural, proneural, and classical. (**a**) miRNA-138/CDK6-involved in proliferation. (**b**) miRNA-139/ZEB1-involved in migration and invasiveness. (**c**) miRNA-203/FGFR1-involved in mesenchymal transition. (**d**) miRNA-218/STAT3-involved in cytokine signaling. (**e**) miRNA-490/TGIF2-involved in EMT. (**f**) miRNA-21/SMAD7-involved in invasion and drug resistance. In survival analysis plots, *p* indicates the P-value. Low and High refer to top and bottom 20% of expressions. In correlation plots, S- indicates the slope of the regression line fitted on the expression data. Complete plots of all target genes that were found for each miRNA are shown in Supplementary Fig. S1 (Supplemental).

In Fig. 2, we demonstrate the survival curves of the selected target gene for each miRNA. We were able to use an online platform that had categorized TCGA_GBM datasets based on four different subtypes of GBM: mesenchymal, neural, proneural, and classical. Most of the selected target genes showed a negative correlation in at least three out of four GBM subtypes. We were not able to get high correlation coefficients owing to data scattering. Plots of all the survival curves and correlations of genes and miRNAs using the TCGA_GBM dataset are shown in Supplementary Figs. S1 and S2. Supplementary Table S4 shows the survival analyses of the 24 dysregulated miRNAs from the TCGA_GBM data. Among the six miRNAs, miRNA-21 was the only miRNA that showed a *p*-value < 0.05. Univariate and multivariate Cox regression results are also shown in Supplementary Table S5 for the six selected genes.

### In vitro therapeutic evaluation in GBM cells of different phenotypes

#### Initial screening for therapeutic effects of miRNAs in U87-MG and T98g GBM cell lines

**Figure 3:**
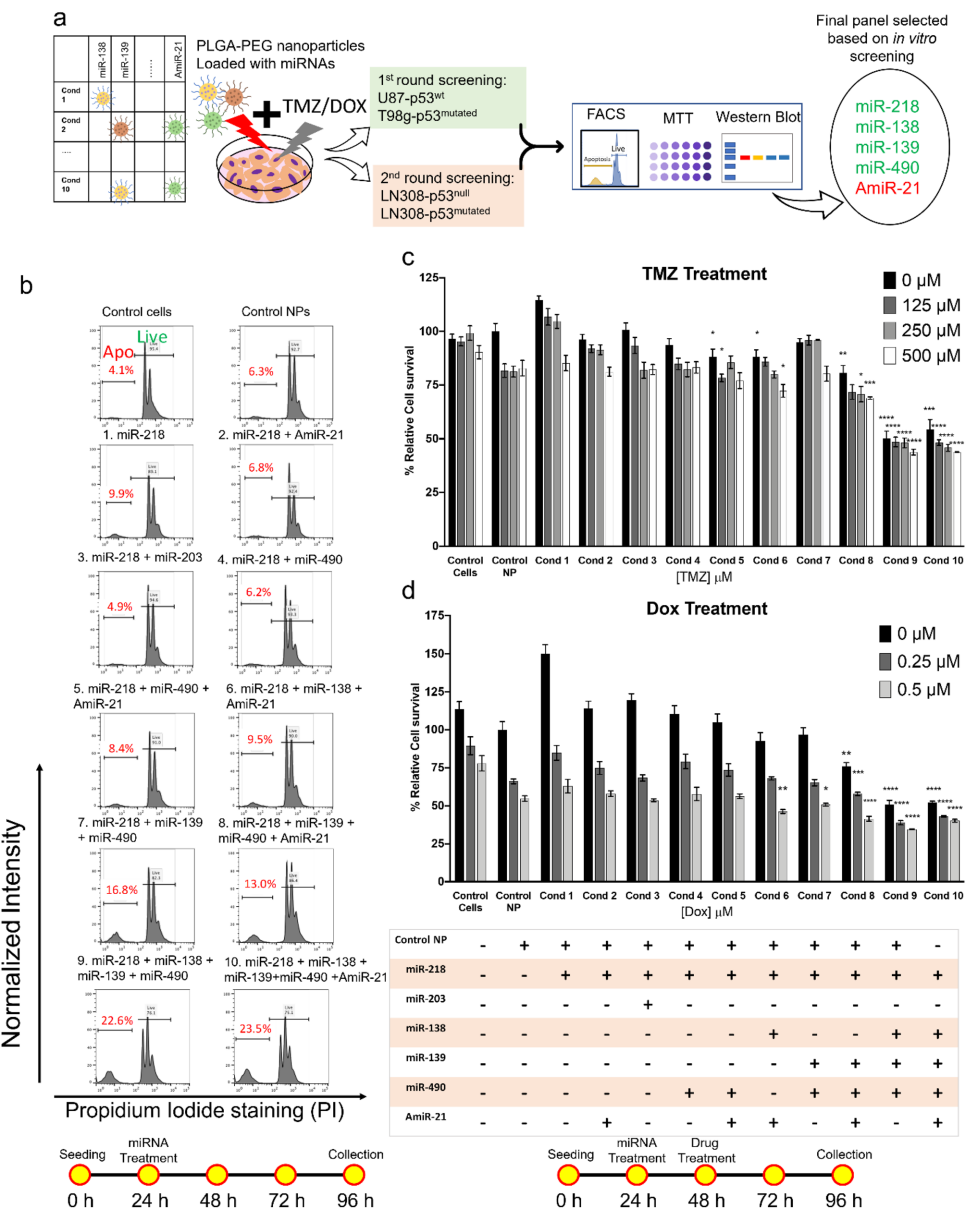
Design and validation of different combinations of miRNAs and their chemosensitizing effects on GBM cells in combination with TMZ or DOX. (**a**) miRNA loaded PLGA-PEG nanoparticles were synthesized using W/O/W double emulsion method optimized in our lab^51^. The miRNAs were then tested in different GBM cell lines. MicroRNA combinations were tested using two different assays: PI staining based FACS to estimate apoptotic population and cell cycle analysis, MTT assay to find the cell proliferation and viability, and western blotting assay to find the expression of selected target genes. (**b**) PI staining based FACS analysis results showing the increase in apoptotic populations after treatment by different combinations of miRNAs as compared to control cells, and cells treated with NPs alone. Numbers shown in red fonts in each subplot are the apoptotic (Apo) populations of the cells as a percentage of total number of cells. (**c**) MTT assay results of cells pretreated with miRNAs and treated with different concentrations of TMZ. (**d**) MTT assay results of cells pretreated with miRNAs and treated with different concentrations of DOX. The table shows the presence or absence of each miRNA in different treatment conditions. Results are expressed as a percentage of survival compared to cells treated with control NPs. All the experiments were carried out on U87-MG cells (repeated three times by independent experiments with triplicates in each experiment). Corresponding timeline for each experimental study is shown below as a schematic workflow. Error bars show standard deviations. Significance levels are shown in the right margins where, *represent *p*-value < 0.05, **represent *p-*value < 0.01, ***represent *p-*value < 0.001, ****represent *p-*value < 0.0001. Asterisks show significant difference compared to control NPs of the corresponding dose.

After identification of miRNAs with target genes involved in signaling pathways related to GBM therapy, we selected different combinations of these miRNAs to investigate the combination that results in better treatment outcome. The selected final six miRNAs were grouped into ten different combinations to cover all four therapeutic pathways that we selected for GBM therapy. We treated GBM cells using different in vitro assays in accordance with workflow in Fig. 3a. We performed FACS and MTT assays to evaluate the apoptotic population and growth inhibition in GBM cell lines. Treatments were performed with and without DOX or TMZ. Characterization of miRNA loaded NPs are shown in Supplementary Fig. S3. We tested the toxicity of NPs by treating the cells with control NPs (Fig. 3b). Incubation of cells using control NPs for three days did not induce a significant increase in the apoptotic population (4.14% for control cells versus 6.36% for control NPs). We treated cells by designing different combinations of miRNAs to cover all four pathways that we introduced earlier (Supplementary Table S6). The results of U87-MG cells treated with different combinations of miRNAs revealed some selective combinations with a significant increase in the apoptotic cell population (Fig. 3b). Specifically, we observed a significant increase in the apoptotic population for conditions #9 and #10 as compared to control NPs (~ 6% control NP versus 23%).

After baseline apoptotic evaluation of miRNA alone therapy, we pretreated U87-MG cells with each miRNA condition for 24 h before subsequent treatment with different concentrations of TMZ or DOX. We diluted the drugs in 100 μL of DMEM to reach the desired concentrations of 125 μM, 250 μM, and 500 μM for TMZ, and 0.125 μM, 0.25 μM, and 0.5 μM for DOX per well. Figure 3c,d show the relative cell viability measured by using MTT assay for cells treated with TMZ or DOX, respectively. All the conditions were normalized to control NP without drug treatment. The MTT assay revealed that incubation of cells with TMZ alone did not show any impact on the viability in concentrations below 500 μM. We observed ~ 10% reduction in cell viability upon incubation of cells with 500 μM TMZ for 48 h. The cells pre-sensitized with miRNAs in conditions #9 and #10 and treated with 500 μM TMZ resulted in the lowest cell viability among all different combinations, which was about 50% for both combinations.

The PI staining based FACS analysis showed that treatment with TMZ alone did not cause any dose-dependent increase in apoptosis. In contrast, we observed significant changes in cell cycle arrest when compared to cells treated with control NPs (Supplementary Fig. S4 and Supplementary Table S7). Similarly, we noticed a significant decrease in cell proliferation when we observed them under a phase contrast microscope. Notably, when we treated the cells with two consecutive doses of 250 μM of TMZ after pretreatment with different combinations of miRNAs, we observed significant changes in cell cycle arrest (Supplementary Fig. S5 and Supplementary Table S8). Cells treated with TMZ alone showed ~ twofold higher cells at G2 phase than in cells treated with control NPs.

On the other hand, DOX alone treatment resulted in a dose-dependent inhibition of cell viability in U87-MG cells. Condition #9 pre-treated with 0.5 μM DOX showed the lowest cell viability among all different conditions, which was around 40%. When cells were treated with 0.5 μM DOX, most of the miRNA treated conditions showed ~ 1.5-fold higher apoptosis than control conditions (Supplementary Fig. S6 and Supplementary Table S9). Based on our initial screening using U87-MG cells, condition #9 (miRNA-218, miRNA-138, miRNA-139, and miRNA-490) and #10 (miRNA-218, miRNA-138, miRNA-139, miRNA-490, and antimiRNA-21) showed the highest apoptotic population and lowest cell viability. We then treated T98g cells with these two conditions and compared to control and control NP treated cells with TMZ and DOX drugs. Condition #9 had a higher apoptotic effect on T98g cells than condition #10. A suboptimal dose of DOX significantly increased the apoptotic population compared to control NPs (~ 8% versus 17%), but as was the case for U87-MG cells, T98g did not show a significant increase in apoptosis after TMZ treatment (Supplementary Fig. S7).

**Figure 4:**
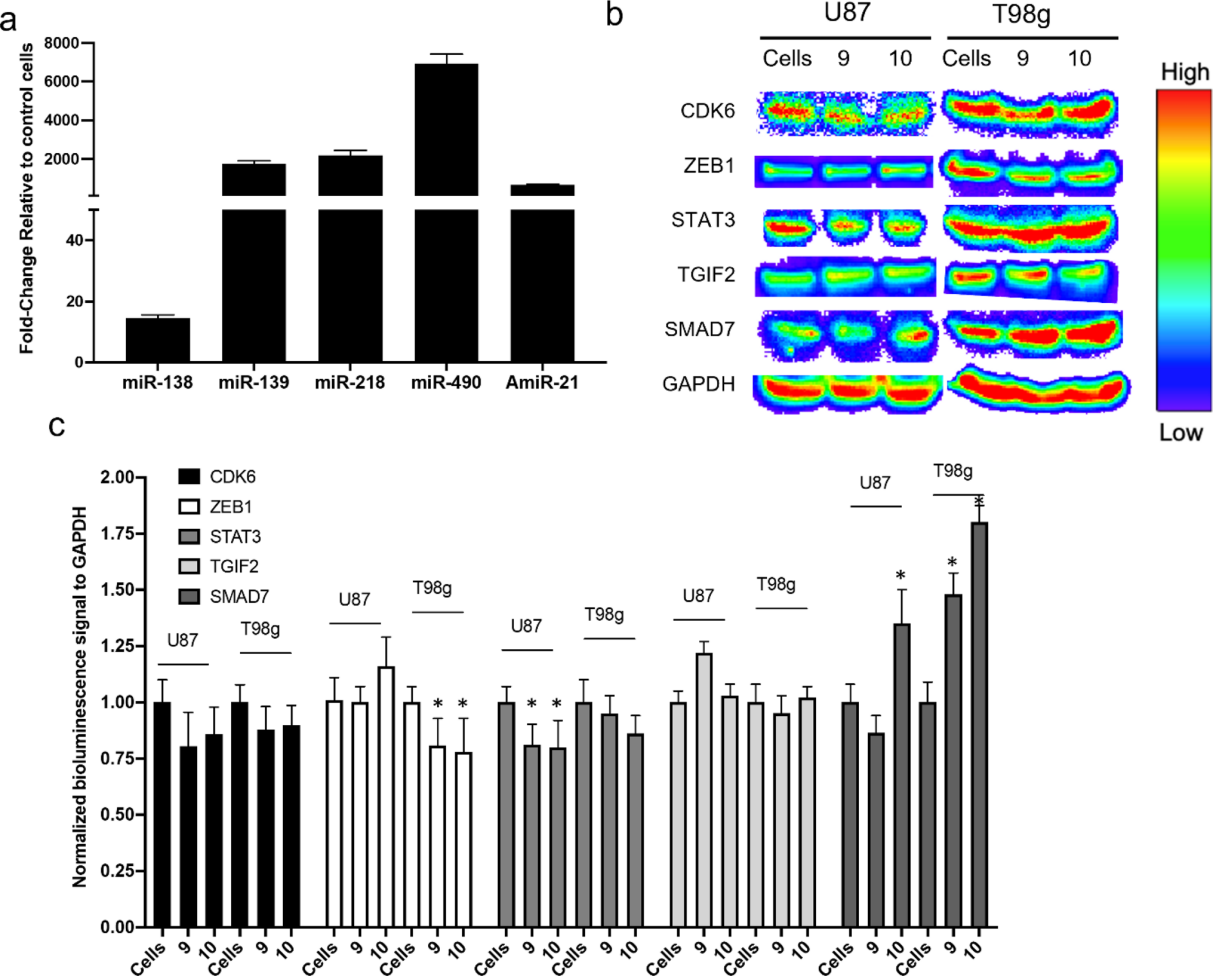
Effect of delivery of miRNAs to downstream target genes. (**a**) Quantitative PCR results showing in vitro delivery of miRNA loaded PLGA-PEG NPs to U87-MG cells. (**b**) Western blotting assays to show the effects of downstream mechanisms of the selected target genes. (**c**) Normalized bioluminescence signals from western blotting assays. The error bars represent the standard deviations of the data. Significance levels are shown in the right margins where, *represent *p*-value < 0.05. Asterisks show significant difference compared to control cells of the corresponding cell line. Cells = control cells without treatment, 9 = cells treated with condition #9, 10 = cell treated with condition #10.

To quantify the level of miRNA delivery in vitro, we treated the U87-MG cells with miRNAs shown in condition #10, and quantified the delivered miRNAs levels by qRT-PCR and compared it in relation to control cells. qRT-PCR analysis showed a significant delivery of miRNAs to U87-MG cells (Fig. 4a). Particularly, miR-490 had the highest fold-change increase after delivery of miR-490 mimic. We also tested the change in the target genes expression levels after treatment with miRNAs in condition #10 (Fig. 4b). We expected an inverse relationship between the expression levels of target genes with the miRNAs. Quantification of western blotting signals revealed that the expression level of SMAD7, the target gene of miR-21, had significantly increased in T98g cells. In contrast, STAT3, the target gene of miR-218, showed significant decrease in U87 cells upon transfection. Also, ZEB1, the target gene of miR-139, had significantly increased in T98g cells. CDK6 also showed an inverse change after treatment with miR-138, but the change in the expression was not statistically significant (Fig. 4c). Whole western blotting membranes that show the location of each band are shown in Supplementary materials (Fig. S8).

#### MiRNA and TMZ as a combination therapy in GBM cells with different p53 phenotypes

**Figure 5:**
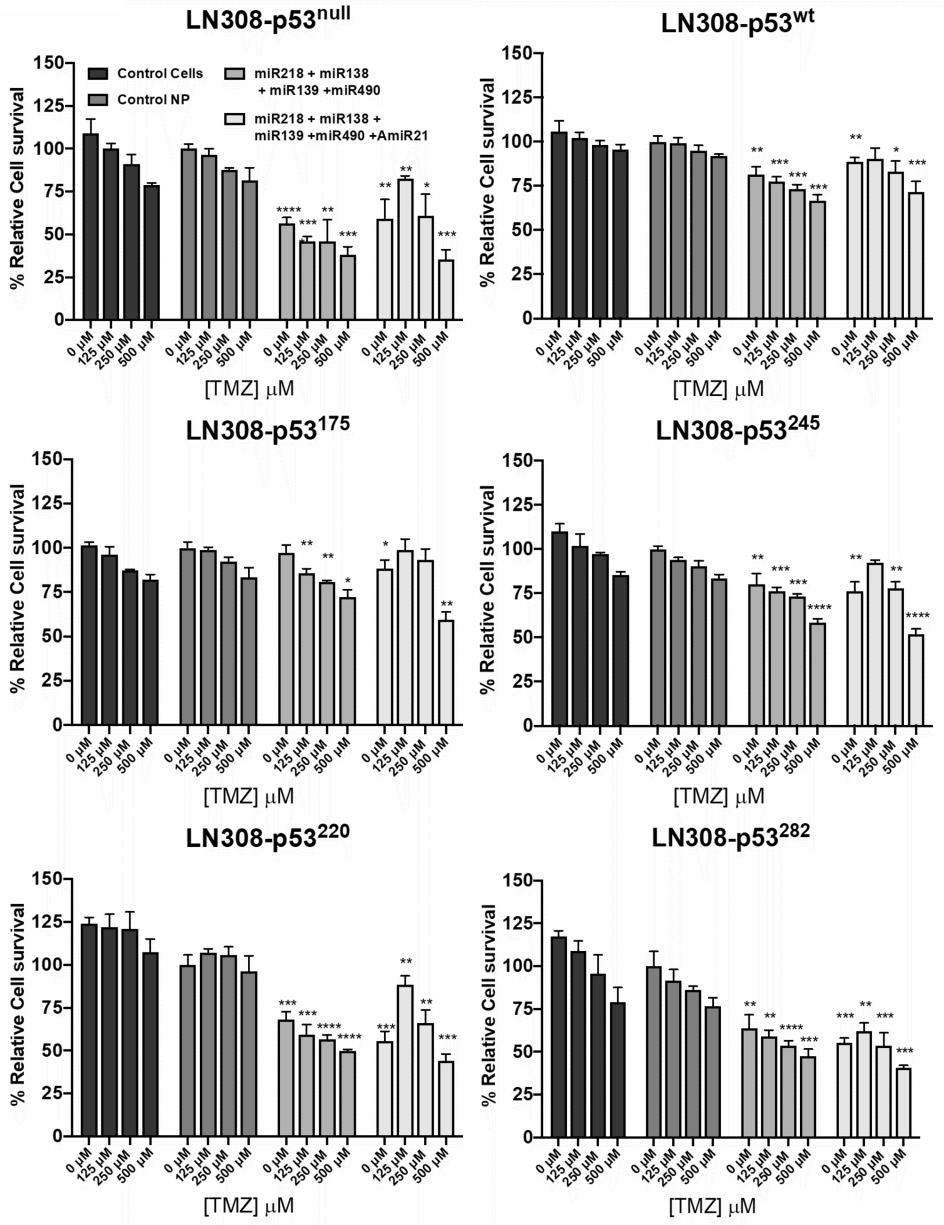
MTT and western blotting assays on LN308 cell line with different p53 variant phenotypes. MTT assay to show the cell viability of LN308 cell lines engineered to express structurally mutant p53 proteins. Each condition was repeated in triplicates. Error bars show standard deviations. Asterisks show significant difference compared to cells treated with control NPs of the corresponding dose where, *represent *p*-value < 0.05, **represent *p-*value < 0.01, ***represent *p-*value < 0.001, ****represent *p-*value < 0.0001.

To further evaluate the effect of miRNAs and TMZ as a combination therapy, we used LN308 GBM cells engineered to express different p53-mutant variants. We tested combinations of miRNAs in conditions #9 and #10 on these cells. Since mutation in p53 gene is very common in GBM and responsible for drug resistance, studying these clinically important mutants in a cell line with a single genetic background is important. Hence, we used Ln308 cells with p53-null background to engineer using respective p53-mutants for the study. MTT assay results after pretreatment with miRNAs and following treatment using 250 μM TMZ (consecutive doses) are shown in Fig. 5. We found that in the absence of p53 expression (LN308-p53^null^), both treatment conditions (#9 and #10) effectively reduced the cell viability. In the case of cells expressing wild type p53 (LN308-p53^wt^), TMZ/microRNAs combination treatment resulted in an improvement of about 20% compared to cells treated using control NPs. We achieved better treatment outcome from cells expressing p53 with mutations at amino acid positions 220, 245 and 282, and treated using miRNAs in condition #10 (around 10% less cell viability). The outcome was greater for condition #10 with higher doses of drugs (DOX or TMZ) compared to the same with condition #9.

#### Therapeutic evaluation of rationally identified miRNAs (miR-138, miR-139, miR-218, miR-490, and AmiR-21) in combination with TMZ in vivo in U87-MG tumor xenografts

**Figure 6:**
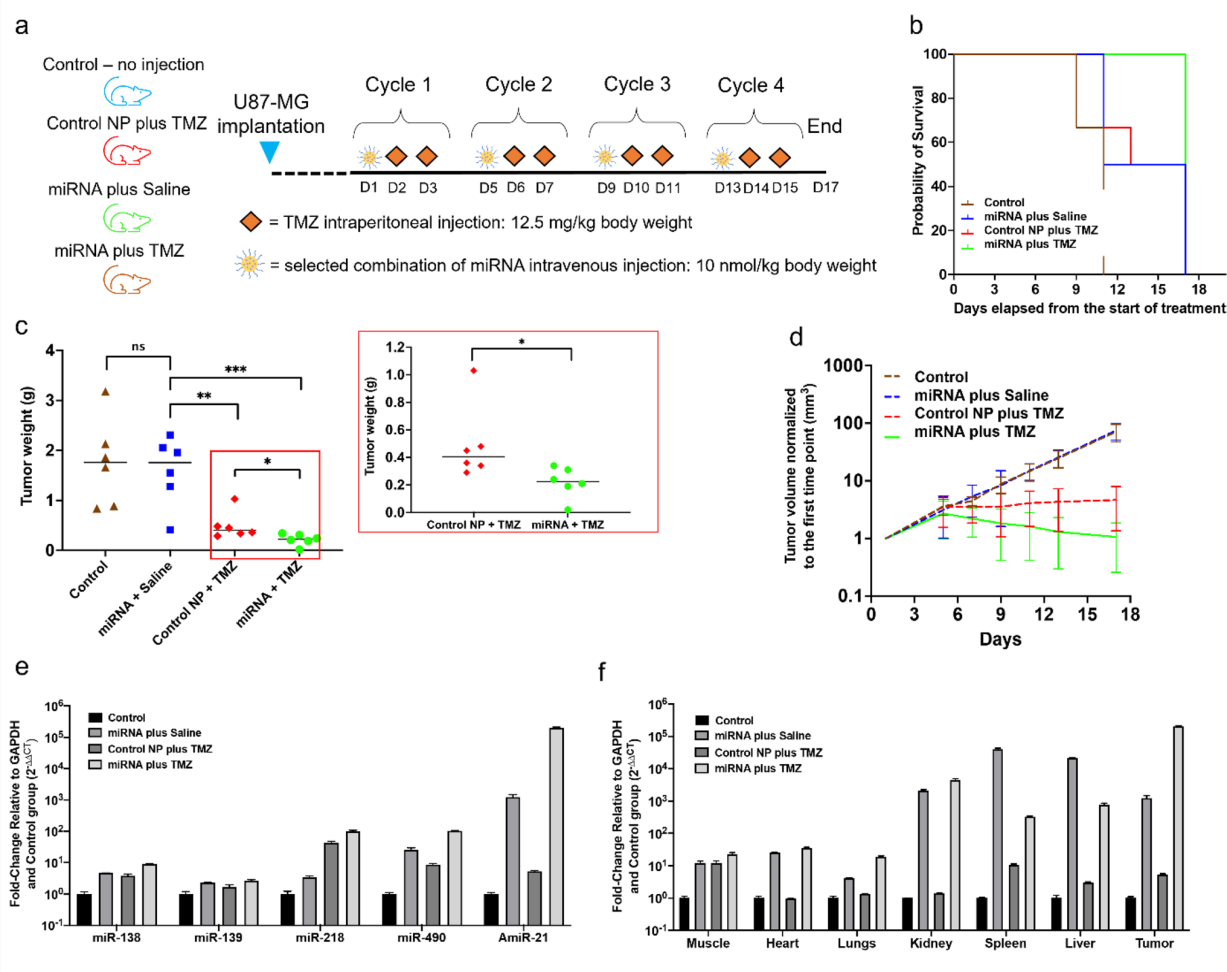
In vivo evaluation of the delivery and chemosensitizing effect of the panel of miRNAs (miR-138, miR-139, miR-218, miR-490, and Amir-21) followed by TMZ in U87-MG xenografts in mice. (**a**) Schematic of in vivo workflow. (**b**) Survival curves of the mice at different treatment group (number of tumors, N = 6–8 per group). (**c**) Tumor weight at the terminal point for mice in different treatment groups. The insert shows the last two groups. (**d**) Estimated tumor volume in different treatment groups. Since some of the mice were sacrificed earlier, all the curves except miRNA plus TMZ were estimated by fitting an exponential growth model to data. (**e**) Biodistribution of miRNA-138, miRNA-139, miRNA-218, miRNA-490, and antimiRNA-21 in tumors of mice in different treatment groups. (**f**) Biodistribution of antimiRNA-21 in different organs of mice in different treatment groups.

We treated the animals for four cycles of PLGA-PEG-NPs loaded with miRNAs (miR-138, miR-139, miR-218, miR-490, and AmiR-21) using the TMZ treatment schedule shown in schematic Fig. 6a. All animals of the group that received miRNA plus TMZ survived beyond four cycles of treatment. In contrast, the control group and the miRNA only group were sacrificed earlier owing to tumor volume reaching above the allowed limit (Fig. 6b). We sacrificed one animal in the TMZ treatment group earlier because of major weight loss. The final tumor weight was not significantly different for the control (average ± SD = 1.76 ± 0.87) and the miRNA alone (1.59 ± 0.69) groups, but it was significantly different between those cohorts and the groups receiving control NP plus TMZ (0.49 ± 0.27) and miRNA plus TMZ (0.22 ± 0.11) (Fig. 6c). We observed a significant difference in the average tumor weight at the final time point between control NP plus TMZ and miRNA plus TMZ groups (*p*-value < 0.05) (Fig. 6c). We also tested this after removing the outliers in the groups, and the results remained significant (*p*-value < 0.05). We obtained the tumor growth plots by fitting exponential growth curves to the corresponding data points for each group and continuing that until the last date of the study. This allowed us to project the tumor growth in groups that had to be terminated earlier (Fig. 6d). Mice in control and miRNA groups had almost similar growth curves, while tumor growth in the control NP plus TMZ group reached a plateau. We observed a significant reduction in tumor size in the miRNAs plus TMZ group. Even though the tumor sizes in the miRNA plus TMZ group continued to show a reduction in volume, we sacrificed the mice after four cycles of treatment to perform ex vivo analysis for miRNA delivery.

We quantified the levels of delivered miRNAs to the tumors using qRT-PCR analysis (Fig. 6e), and to the major organs (Fig. 6f) using antimiRNA-21 as a representative RNA. The most downregulated miRNAs had a higher-fold change in the miRNA plus TMZ group than the other conditions. MiRNA-218 showed approximately an 80-fold higher change in groups that received TMZ treatment, which might suggest that TMZ affects the expression of this miRNA in GBM.

## Discussion

Tumor resistance in GBM is in part attributed to the aberrant expression of miRNAs and their target genes^22,23^. A couple of the setbacks in moving miRNA therapies from bench to bedside are the lack of effective delivery methods and the absence of a set of high priority intracellular targets^24^. Here, we developed a more rational approach by initial determination of different combinations of abnormally expressed miRNAs in GBM after analysis of various expression databases. The panel of miRNA-138, miRNA-139, miRNA-218, miRNA-490 and antimiRNA-21 showed the lowest cell survival and highest apoptosis in vitro. We further evaluated this panel in vivo. Differentially expressed miRNAs with therapeutic roles in GBM were also identified in previous studies^25–28^. miR-10b (up), miR-129-1-3p (down), miR-139 (down), miR-124 (down), miR455 (up) were commonly identified amongst the top dysregulated miRNAs in GBM vs. normal brain tissue.

Among the selected miRNAs, miRNA-21 was the only upregulated miRNA identified from our screen. We and others had shown that miRNA-21 inhibition is an effective therapeutic approach to improve GBM treatment^29–31^. MiRNA-21 is associated with several downstream pathways that are involved in cell proliferation, kinase activity, drug resistance, and apoptosis. Overexpression of miRNA-21 decreases the apoptosis of GBM cells after treatment with TMZ by targeting apoptosis inducing proteins (PTEN, PDCD4, etc.,)^32^. MiRNA-218 is specifically known for glioma invasion, and its downregulation in GBM is associated with genes encoding for RTK signaling pathways^33^. Based on our findings, miRNA-218 alone does not significantly improve TMZ- or DOX-mediated apoptosis in GBM cells. This could be because miRNA-218 is mainly involved in invasion and metastasis. MiRNA-138 is a tumor suppressor and it inhibits GBM tumorigenicity. Studies have shown that *EZH2* (which contributes to tumor proliferation), and *CDK6*, a critical regulator of cell cycle transition in G1/S, are both upregulated in GBM and are direct target genes of miRNA-138^34^. Our data showed that among different targets only *CDK6* showed a negative correlation between miRNA-138 and gene expression in all GBM subtypes. The other three targets fail to at least show a negative correlation in the classical subtype. The role miRNA-139 in invasion and metastasis was highlighted through regulation of *NOTCH1*^35^, and *ZEB1*^36^. MiRNA-490 is known as a tumor suppressor in GBM, and it is significantly downregulated in glioma cell lines. Besides the target genes that we investigated for miRNA-490 in this study (*NOTCH1* and *TGIF2*), recent studies have shown that this miRNA inhibits the oncogenic protein, HMGA2^37^, and a telomere maintaining program protein, TERF2^38^.

We predominantly focused on TMZ and DOX in this study. TMZ is a prodrug, which loses its functional effect rapidly in the systemic circulation. It requires a metabolic reaction to become active. In addition, TMZ is stable only for a short time in cell culture medium with neutral pH, hence its prolonged therapeutic action is limited. DOX is a potential anticancer drug that is not in clinical use against GBM owing to limitations in passing the BBB, as well as its high toxicity^39^. Novel drug delivery solutions such as PLGA-PEG-NPs^40^, cell membrane coated nanoparticles^41,42^, and 3D scaffolds^43^ are under investigation. It would be advantageous to patients to achieve therapeutic effects at lower drug doses to reduce their side effects. In this study, we observed a significant decrease in GBM cell survival when tested at suboptimal doses of DOX after sensitizing the cells using miRNAs. TMZ is known to induce cell cycle arrest in G2/M^44^, though, G1 cell cycle arrest was reported when GBM cells were treated with some miRNAs and TMZ in vitro^45^. Yet, we did not observe any significant change in cell cycle arrest with single dose TMZ treatment. Indeed, the independent beneficial effects of miRNA-21^31,46^, miRNA-138^47^, miRNA-139^48^, and miRNA-218^49^ with concomitant TMZ treatment were shown in previous studies.

Our in vivo results show that delivery of the combination of miRNAs without any drug administration does not improve tumor suppression. However, when these miRNAs are delivered prior to drug administration, the tumor growth starts declining as early as six days after the start of treatments (after the first cycle of combinational treatment). We observed a continuous tumor growth in mice receiving miRNAs alone, while the group receiving TMZ had reached a plateau in tumor growth without any further decline in size. Moreover, in some of the animals that were treated with TMZ alone, we observed a significant weight loss. In contrast, the group that received combinational treatment had a better therapeutic effect without any toxicity. The histological analysis shows that the NPs did not cause any toxicity to tissues (Supplementary Fig. S9).

In conclusion, our findings expand the list of miRNA candidates that are potential therapeutic targets to increase the chemosensitivity of GBM cells. Pre-treatment of cells with a rationally selected panel of five miRNAs induced apoptosis when accompanied by sub-toxic doses of subsequent TMZ and DOX treatment. PLGA-PEG-NPs are non-toxic vehicles to deliver miRNAs into the cells, with high intracellular delivery efficacy. A more optimal and clinically relevant dosing schedule using orthotopic tumors in immunocompetent mouse models will be necessary in future studies that also address the immunomodulatory role of our identified panel of therapeutic miRNAs.

## Methods

### In silico analysis

#### Ethical consent regarding the use of data from TCGA and GEO

We obtained all patients’ data from publicly available databases (TCGA and GEO). There was no need for ethical approval as all data in this study were downloaded from public databases, and the data processing met their publication guidelines. The active link to access each source is given in the supplementary information under data and code availability.

#### Identification of dysregulated GBM-specific miRNAs

To select a combination of miRNAs that sensitize GBM to chemotherapy, we analyzed expression datasets of human GBM from GEO (GSE25631 and GSE90603) and TCGA. Those miRNAs with a false discovery rate (FDR) adjusted *p*-value of < 0.05 and absolute log2‐fold change (|log_2_FC|) of > 1 were considered significant in each dataset. We identified the commonly deregulated miRNAs in these three datasets.

#### Identification of the target genes of dysregulated miRNAs in GBM

We first identified the validated target genes of dysregulated miRNAs using TargetScan and miRTarBase 8.0. Next, we applied three additional criteria to narrow down the number of target genes associated with each miRNA in GBM. These criteria were: (1) the validated gene must have been differentially expressed in GBM versus normal brain tissue (analyzed by GEPIA^50^), (2) there should be a negative correlation between the target gene and miRNA expressions, and (3) the target genes must have been validated by published literature to be involved in one of the four aforementioned pathways in GBM. We eliminated from the list those target genes that did not meet these criteria. Details of each criterion are in the Supplementary Methods.

#### Kaplan–Meier survival curves analysis

We used the TCGA-GBM dataset for survival analyses. In univariate analyses, we tested the significance of survival in different settings including all the patients, and separating patients with and without MGMT promoter methylation, and with and without TMZ treatment. Kaplan–Meier survival analyses were performed for the top 20% and the bottom 20% expression groups. We also performed univariate and multivariate cox regression on the main target genes.

### In vitro therapeutic evaluations in GBM cells

#### MiRNA design and synthesis

Five miRNA mimics and one antisense miRNA were synthesized by the Protein and Nucleic (PAN) Facility at Stanford. The sequence of each miRNA can be found in the Supplementary Methods. We synthesized PLGA-PEG-NPs based on a previously reported protocol and loaded them with miRNAs in individual batches^51^.

#### Cell culture and transfection

Human GBM cell lines (U87-MG, T98g, and LN308, from ATCC, and LN308 variants engineered in our laboratory) were selected for this study because of their differences in phenotype and expression of p53 gene. LN308 is a known p53-null GBM cell line, and we previously engineered this to express clinically important p53 mutations at different sites. Specifically, we used the following engineered LN308 cell lines for this study: p53^wt^, p53^175^, p53^220^, p53^245^, and p53^282^.

#### Cell culture and treatment for detection of FACS-based apoptosis and for MTT assay

Twenty-four hours prior to miRNA transfection, we seeded the cells in 12- or 96-well plates for FACS and MTT cell viability assays, respectively, to achieve 70% confluency. On the day of transfection, we washed the cells with PBS. Each well was then transfected using miRNA loaded PLGA-PEG-NPs with the total concentration of 0.1 nM miRNAs. The total concentration was balanced with the control PLGA-PEG-NPs. If cells were treated with TMZ or DOX we added the drugs in the corresponding concentrations at the corresponding time. The corresponding timeline for each study is drawn below each graph. Each condition was tested in triplicate.

#### FACS analysis

We fixed the cells in 80% ethanol after collection and stained them with PI (Supplementary Material). We performed the FACS analysis using a Guava analytical system in red-blue emission spectra. We analyzed apoptotic and live cells, as well as the cell cycle data using FlowJo™ software. We expressed the results as the percentage of total cells.

#### Cell viability assay (MTT)

On the day of MTT assay, we added MTT and dissolved the formed violet formazan crystals in DMSO based on the protocol in the Supplementary Material. The absorbance was recorded at 565 nm wavelength for each well. We analyzed the results and compared them with the control condition, which was considered 100%.

#### RNA extraction and RT-PCR analysis

We quantified the delivery of miRNAs in cells (culture study) and tumor tissue and organs harvested from mice. We lysed cells and tissues and extracted total RNA from them using the mirVana miRNA extraction kit, by following the manufacturer’s protocol. After quantification, 200 ng of total RNA equivalent was reverse-transcribed using RT primers (TaqMan MicroRNA Assays) using a reverse-transcription kit (Applied Biosystems) to produce the corresponding cDNA. qRT-PCR was performed in CFX96 Touch Real-Time PCR system (BioRad). The expression of miRNAs was calculated using the 2^−ΔΔCT^ method and normalized by GAPDH. We purchased all the primers from Thermo Fisher Scientific, except antimiRNA-21 that we custom designed.

#### Immunoblot analysis

We measured the protein expression levels of genes in response to miRNA treatments using western blot analysis (Supplementary Material). We incubated the membrane with corresponding primary antibody for each of the target proteins listed at 4 °C overnight following the dilutions of: SMAD7 (1:500, SCBT, sc365846), CDK6 (1:500, SCBT, sc7961), ZEB1 (1:1000, CST, 70512S), STAT3 (1:500, SCBT, sc8019), TGIF2 (1:500, SCBT, sc81989), and GAPDH (1:2000, CST, 5174S), respectively. We washed the membrane in PBST and then incubated with horseradish peroxidase conjugated anti-rabbit or anti-mouse secondary antibody (1:5000) for 1 h at room temperature in a shaker. We further washed the membranes and imaged them in an IVIS-Lumina imaging system. We used the membranes processed using GAPDH antibody (Cell Signaling Technologies) as an internal control.

### In vivo experiments in GBM mouse model

All animal experiments were approved by the Stanford University Administrative Panels on Laboratory Animal Care (protocol code 33144 and date of approval 11/02/2021). All experiments were performed in accordance with relevant guidelines and regulations. We also followed the recommendations in the ARRIVE guidelines. We used nude (Nu/Nu) mice aged 4–6 weeks. Mice were implanted with 5 × 10^6^ U87-MG GBM cells into their left and right flanks. Tumors were allowed to grow until they reached 2–3 mm in diameter. We randomized the mice into 4 groups (number of tumors, N = 6–8 per group). Control mice did not receive any treatment, the TMZ (control NP plus TMZ) treated group received control NPs and TMZ, the miRNA group (miRNA) received the panel of miRNAs shown in condition #10, and the miRNA plus TMZ group received the combination of the panel of miRNAs and TMZ. We treated mice with intravenous injection of NPs followed by two consecutive doses of TMZ intraperitoneally in 10% PEG-400 in the subsequent 2 days, with one day of no treatment. We repeated this cycle four times for the following 16 days before sacrificing the mice for collecting the organs for ex vivo analysis. We recorded each mouse weight and tumor volume for the following 16 days until the end of treatment. We sacrificed all animals on Day 17 and collected the organs and tumors for ex vivo quantification of miRNA delivery, therapeutic effect, and toxicity using histological analysis. We checked the biodistribution of antimiRNA-21 in all organs and the delivery of all miRNAs in tumors using TaqMan real-time qRT-PCR, as described earlier.

### Statistical analysis

We performed statistical analyses using Prism (GraphPad) and student’s unpaired t-test. *p*-values < 0.05 were considered statistically significant. Differences in survival curves were determined using the Log-Rank test. All error bars represent standard deviations. *, **, ***, **** represent *p*-value < 0.05, < 0.01, < 0.001, < 0.0001, respectively.

## Supplementary Information

Supplementary Information.

## Data Availability

The datasets for microarray analysis were available through the Gene Expression Omnibus Series (https://www.ncbi.nlm.nih.gov/geo/) number GSE25631 and number GSE90603. We analyzed the deregulated miRNAs using the GEO2R online tool (https://www.ncbi.nlm.nih.gov/geo/geo2r/). The Cancer Genome Atlas data were available in https://portal.gdc.cancer.gov, and the data, including the miRNA expression levels and patient identification numbers were downloaded from the FireBrowse platform (http://firebrowse.org/) and analyzed in R. We also identified the predicted target genes of miRNAs by using miRTarBase (http://mirtarbase.cuhk.edu) and by searching for the keywords containing the miRNA name and “GBM” in Pubmed. To find which of the miRNAs target genes were differentially expressed, we used GEPIA (http://gepia.cancer-pku.cn), an online tool for analyzing TCGA and GTEx datasets. The TCGA_GBM survival curves were analyzed using OncoLnc (http://www.oncolnc.org), a TCGA data portal with an emphasis on survival analysis.
