# Supplementary material for: A rationally identified panel of microRNAs targets multiple oncogenic pathways to enhance chemotherapeutic effects in glioblastoma models
Source: Sci Rep. 2022 Jul 14;12:12017. doi: 10.1038/s41598-022-16219-x (PMC9283442; doi:10.1038/s41598-022-16219-x)
Supplement: Supplementary file 1 — Supplementary Information. [file 41598_2022_16219_MOESM1_ESM.docx]

**A Rationally Identified Panel of MicroRNAs Targets Multiple Oncogenic Pathways to Enhance Chemotherapeutic**

**Effects in Glioblastoma Models**

**Negar Sadeghipour, Sukumar Uday Kumar, Tarik F Massoud*, and**

**Ramasamy Paulmurugan***

Molecular Imaging Program at Stanford (MIPS), Stanford University School of Medicine,

Stanford, CA, USA. (N.S, S.U.K, T.F.M, R.P)

The Canary Center at Stanford for Cancer Early Detection, Stanford University School of

Medicine, Palo Alto, CA, USA. (N.S, S.U.K, R.P)

**Supplementary Methods**

***In silico analysis***

**Ethical consent regarding the use of data from TCGA and GEO.** There was no need for ethical approval as all data in this study were downloaded from public databases, and the data processing met their publication guidelines.

TCGA: <https://www.cancer.gov/about-nci/organization/ccg/research/structural-genomics/tcga>, GEO: (<https://www.ncbi.nlm.nih.gov/geo/>).

**Identification of GBM-specific miRNAs for therapeutic applications.** To select a combination of miRNAs that sensitize GBM to chemotherapy, we analyzed expression datasets of human GBM from GEO (GSE25631 and GSE90603) and TCGA. GSE25631 contained data on 82 surgical specimens of primary GBM and five normal brain tissue specimens; and GSE90603 contained 16 GBM specimens and seven normal brain tissue specimens. The GSE25631 dataset was based on the GPL8179-Illumina Human v2 MicroRNA expression bead chip, and the GSE90603 dataset was based on GPL21572-Affymetrix Multispecies miRNA-4 Array. We used the GEO2R online tool to identify the differentially expressed miRNAs in human cancers versus normal tissues using the limma package in R. Those miRNAs with a false discovery rate (FDR) adjusted p-value of < 0.05 and absolute log2‐fold change (|log_2_FC|) of > 1 were considered significant in each dataset.

To improve the statistical significance and reliability of our analysis, we included miRNA expression data from the TCGA_GBM dataset. This contained 196 cancer samples and 10 adjacent normal brain tissue controls. We obtained the TCGA_GBM dataset through the Genomic Data Commons (GDC) portal. We first transformed the miRNA expression levels measured by reads per million miRNAs mapped into log2. We then used R statistical analysis tool to identify the differentially expressed miRNAs in the TCGA dataset. As we had established for the GEO datasets, those miRNAs with a false discovery rate (FDR) adjusted p-value of < 0.05 and absolute log2‐fold change (|log_2_FC|) of > 1 were considered significant in the ­TCGA_GBM dataset. We identified the commonly deregulated miRNAs in these three datasets.

**Identification of therapeutic target genes of dysregulated miRNAs in GBM.** We first identified the validated target genes of dysregulated miRNAs using miRTarBase 8.0. This platform uses text mining techniques to pre-screen research articles to find miRNA-target interactions (MTIs), which were then processed manually and reported. For each miRNA, we considered the target genes that had been validated using reporter assays, western blot, and qPCR. These criteria were (1) the validated gene must have been differentially expressed in GBM versus normal brain tissue, (2) there should be a negative correlation between the target gene and miRNA expressions, and (3) the target gene must have been validated by published literature to be involved in one of the four aforementioned therapeutic pathways in GBM. We eliminated from the list those target genes that did not meet these criteria. We identified the expression of the target genes in GBM and normal brain tissues from the online interactive web server, GEPIA (Gene Expression Profiling Interactive Analysis). In GEPIA, the differentially expressed data were obtained from 163 GBM samples and 207 normal brain samples. Genes with FDR p-value of < 0.05 and absolute log2‐fold change (|log_2_FC|) of > 1 were considered significant in GEPIA. We found the correlation between the expression of miRNAs and their target genes for four different GBM subtypes in TCGA_GBM using the platform Betastasis (used for interactive visualization of selected cancer datasets). The expected correlation was a line with a negative slope, and we eliminated from the list those target genes that did not exhibit this correlation. To find information regarding GBM target genes, we investigated them in the search engines (using keywords of “glioblastoma” or “GBM”, and the name of the miRNA), and reviewed the related scientific articles in the published literature. If any of these target genes were known to be involved in one of four pre-determined therapeutic pathways (apoptotic and growth signaling, invasion and metastasis, cytokine signaling, and stemness), we selected them for further bioinformatics analysis.

**Kaplan-Meier survival curves analysis.** To identify the association between the survival of GBM patients and the shortlisted miRNA expression and the target gene, we performed survival analyses. We explored the survival analysis of each target gene in the TCGA_GBM dataset. In all analyses we divided patients into high expression and low expression groups based on the lowest and highest (20% to 20% cutoff or 33% to 33% cutoff). We used the Kaplan-Meier estimation method and reported two-sided log-rank p-values. The p-values <0.05 were considered significant. In general, GBM patients have a relatively short survival time, and as a result, there was infrequent observance of significantly different survival times between the high and low expression groups.

**Data and code availability.** The datasets for microarray analysis were available through the Gene Expression Omnibus Series (<https://www.ncbi.nlm.nih.gov/geo/>) number GSE25631 and number GSE90603. We analyzed the deregulated miRNAs using the GEO2R online tool (<https://www.ncbi.nlm.nih.gov/geo/geo2r/>). The Cancer Genome Atlas data were available in <https://portal.gdc.cancer.gov>, and the data, including the miRNA expression levels and patient identification numbers were downloaded from the FireBrowse platform (<http://firebrowse.org/>) and analyzed in R. We also identified the predicted target genes of miRNAs by using miRTarBase (<http://mirtarbase.cuhk.edu>)and by searching for the keywords containing the miRNA name and “GBM” in Pubmed. To find which of the miRNAs target genes were differentially expressed, we used GEPIA (<http://gepia.cancer-pku.cn>), an online tool for analyzing TCGA and GTEx datasets. The TCGA_GBM survival curves were analyzed using OncoLnc (<http://www.oncolnc.org>), a TCGA data portal with an emphasis on survival analysis.

***In vitro therapeutic evaluations in GBM cells***

**Materials.** We purchased Phosphate Buffered Saline (PBS) (Corning, VA, USA), Dulbecco’s Modified Eagle Medium (DMEM) (Corning, VA, USA), Fetal Bovine Serum (FBS) (Gibco, CA, USA), penicillin/streptomycin (Gibco by Life technologies, USA), Doxorubicin hydrochloride (DOXO, Sigma Aldrich, St. Louis, MO, USA), and Temozolomide (Tocris Bioscience, Bristol, UK). We prepared aqueous stock solutions of various buffers using 18.2 MΩ ultrapure water obtained from a Milli-Q Plus Millipore water filtration system, Dimethyl Sulfoxide (DMSO) (Fisher Scientific, USA), MTT reagent (Life technologies, OR, USA), and Propidium Iodide (Sigma, MO, USA). Carboxy terminal Poly (lactic-co-glycolic) acid–poly (ethylene glycol) conjugate (PLGA-b-PEG) polymer was purchased from Poly SciTech (West Lafayette, IN, USA). Span-80, Polysorbate 80 and Spermidine were procured from Sigma-Aldrich (MO, USA). Syringe ﬁlters of 0.45 µm (Whatman PURADISC 25 AS,PES) were purchased from GE Healthcare (WI, USA) and Centrifugal filters with 100 kDa molecular weight cut-off (MWCO) Membrane were purchased from Millipore (MA, USA).

**MiRNA design and synthesis**. Five miRNA mimics and one antisense miRNA were synthesized by the Protein and Nucleic (PAN) Facility at Stanford. The sequence of each miRNA was as follows: miRNA-218-3P: UUGUGCUUGAUCUAACCAUGU; miRNA-490-: CAUGGAUCUCCAGGUGGGU; AmiRNA-21-: UAGUGAAGCCACAGAUGUA; miRNA-138: AGCUGGUGUUGUGAAUCAGGCCG; miRNA-139: UCUACAGUGCACGUGUCUCCAGU; and miRNA-203: AGUGGUUCUUAACAGUUCAACAGUU.

**MiRNA loaded PLGA-PEG nanoparticle synthesis and dynamic light scattering (DLS).** We synthesized PLGA-PEG-NPs based on a previously reported protocol and loaded them with miRNAs in individual batch. We measured the mean diameter (z- average) of NPs using dynamic light scattering (DLS). For DLS measurements, we diluted the samples in 18 MΩ water, and averaged data from at least three measurements.

**Cell culture and transfection.** Human GBM cell lines (U87-MG, T98g, and LN308 and its variants) were selected for this study because of their differences in phenotype and expression of p53 gene. LN308 is a known p53-null GM cell line, and previously they were engineered in our laboratory to express clinically important p53 mutations at different sites. Specifically, we used the following engineered LN308 cell lines for this study: p53^wt^, p53^175^, p53^220^, p53^245^, and p53^282^. We used these five cell lines together with LN308-p53^null^ for the *in vitro* study. We cultured the cells in DMEM with 10% FBS, 1% streptomycin, and 100 U/mL penicillin and maintained in an incubator with 5% CO_2_ at 37 °C. We passaged the cells every 3-4 days and tested for mycoplasma periodically. Cells were counted using a hemocytometer before plating for experiments.

### Detection of apoptosis by PI staining-based FACS analysis. We suspended cells in DMEM and seeded 1 × 10^5^ cells in 1 mL of media per well of 12-well plates 24 h prior to transfection using miRNA-loaded PLGA-PEG-NPs. On the day of transfection, we washed the cells with 500 μL of PBS. Each well was then transfected using PLGA-PEG-NPs with the total concentration of 0.1 nM of miRNAs. The total concentration was balanced with the control PLGA-PEG-NPs. We monitored the cells for 72 h post-transfection and then collected them for FACS analysis. Each condition was tested in triplicate.

**Treatment with chemotherapeutic drugs (temozolomide or doxorubicin).** For the treatment of cells using chemotherapeutic drugs, we repeated the transfection study as stated above. However, 24 h after the miRNA-loaded NP treatment we added the drugs in different concentrations. We diluted the drugs in 100 μL of DMEM to reach the desired concentrations of 125 μM, 250 μM, and 500 μM for TMZ, and 0.125 μM, 0.25 μM, and 0.5 μM for DOX per well. In experiments where two consecutive doses were delivered, the same concentration of drug was given 24 h after the first dose. We collected the cells for FACS 48 h after the single drug dose or 24 h after the second drug dose for those in consecutive dose experiments.

**FACS analysis.** We collected cultured media from triplicates in 15 mL tubes and washed the cells with 500 μL PBS. The PBS was also collected to ensure all the cells were collected. We then collected the leftover adherent cells using trypsin following neutralization with DMEM. We pelleted the cells by centrifugation at 1000 rpm for 3 min. The supernatant was aspirated, and cells were re-suspended in 500 μL of PBS. We added 2 mL of ice-cold 100% ethanol (to a final concentration of 80% ethanol) while vortexing to ensure the cells did not aggregate. We then kept cells at -20 ℃ for a minimum of 30 min. For FACS analysis (Guava® easyCyte HT), we centrifuged the cells at 1000 rpm for 3 min, aspirated the ethanol, and then washed the cells with 500 μL of PBS and pelleted again. Propidium iodide (PI) staining solution was prepared by mixing 10 μL each of 10 mg/mL PI and RNaseA, and TritonX-100 to a final concentration of 0.01%. We added 500 μL of the staining solution to each condition. After mixing, the cells were placed in the dark for 20 min. We centrifuged the stained cells again, then removed the staining solution and re-suspended in 500 μL of PBS. We performed the FACS analysis using a Guava analytical system in red-blue emission spectra. We analyzed apoptotic and live cells, and the cell cycle data by using FlowJo^TM^ software. We expressed the results as the percentage of total cells.

**Cell viability assay (MTT).** We seeded cells at 5 × 10^3^ cells per well in 100 μL of DMEM in 96-well plates 24 h prior to transfection. On the day of treatment, we washed the cells with 100 μL of PBS and replaced the culture medium with medium containing PLGA-PEG-NPs loaded with the combination of miRNAs that were diluted in DMEM (0.1 nM/well). 100 μL of each condition was supplemented to the cultured media in each well. We treated them after 24 h. We then used serial dilutions of DOX at concentrations of 0, 0.125 μM, 0.25 μM, and 0.5 μM; and TMZ at concentrations of 0, 125 μM, 250 μM, and 500 μM. We had three replicates for each concentration. We aspirated the media 48 h after single dose treatments or 24 h after the second dose of double dose treatments, and added 50 μL of 12.5 mM MTT dissolved in phenol red free DMEM (10% FBS) to each well, and the plates were incubated for 2 h at 37 ℃. After incubation, we carefully removed medium and added 100 μL DMSO to dissolve the violet formazan crystals converted by the mitochondrial enzyme. Using a multi-channel pipette, we pipetted the DMSO up and down several times to completely solubilize the sample. Then we incubated the plates in 37 ℃ in the dark for at least 30 min. The plates were then shaken for 3 s in a plate reader (Tecan Infinite M1000) and absorbance was recorded at 565 nm wavelength for each well. We analyzed the results and compared them with the control condition, which was considered 100%.

**RNA extraction and RT-PCR analysis.** Portions of the tumor tissue and organs harvested from each treatment group of mice were flash frozen and stored at -80 ℃ to quantify the delivery of therapeutic miRNAs. We lysed tissues and extracted total RNA from the tissues using the mirVana miRNA extraction kit, by following the manufacturer’s protocol. The total RNA was checked for purity using a Nanodrop spectrophotometer. After quantification, 200 ng of total RNA equivalent was reverse-transcribed using RT primers (TaqMan MicroRNA Assays, Applied Biosystems) using a reverse-transcription kit (Applied Biosystems, Foster City, CA) to produce the corresponding cDNA. The cDNA synthesis was carried out in a 15 μL reaction volume. qRT-PCR was performed using cDNA (5 ng of RNA equivalent) combined with TaqMan-PCR reagents (primer and probe mix). qRT-PCR was performed by 2 min incubation at 50 °C followed by activation of the DNA polymerase at 95 °C for 10 min, 50 cycles of 95 °C for 15 s, and 60 °C for 60 s in CFX96 Touch Real-Time PCR system (BioRad). The qRT-PCR reaction was carried out in a 20 μL reaction volume. The expression of miRNAs was calculated using the 2^−ΔCT^ method. We purchased all of the primers from Thermo Fisher Scientific, except antimiRNA-21 that we costume designed.

### Immunoblot analysis. We measured the protein expression levels of therapeutic genes in response to miRNA treatments using western blot analysis. Twenty four hours prior to transfection we counted and seeded cells at the density of 2 × 10^5^ cells per well in 6-well plates in 2 mL of DMEM. On the day of transfection, we washed the cells with PBS, and the cultured medium was replaced with DMEM containing the combinations of miRNAs. After 48 h, we collected the cells via trypsinization and pelleted by centrifuging at 5,000 rpm for 5 min. We lysed the cell pellets in RIPA buffer (Pierce® RIPA Buffer, Thermo Scientific) containing 1% protease inhibitor (Halt^TM^ Protease Inhibitor, Thermo Scientific) and 1% EDTA (0.5 M, Gibco) by sonicating three times for 15 s each at 40% amplitude. We centrifuged the lysed cells at 15,000 rpm for 15 min at 4 ℃ and collected the supernatants. We estimated the protein concentration using a BCA protein assay kit (Pierce Chemicals Co, Dallas, TX, USA). The proteins of each sample were normalized to the sample of the lowest concentration by adding RIPA buffer prior to gel loading. We added 25% volume of 4X loading buffer (NuPAGE LDS Sample buffer 4X, Life Technologies Corporation, CA, USA) containing 5% β-mercaptoethanol (Aldrich, Germany) to each sample, and denatured the samples by heating at 95 ℃ for 5 min. We resolved the samples by electrophoresis on a 4-12% pre-cast gradient SDS-polyacrylamide gels (Life Technologies, Carlsbad, CA, USA). We transferred each gel to a nitrocellulose membrane, which was then blocked using PBST containing 5% skim milk powder. We incubated the membrane with corresponding primary antibody for each of the target proteins listed earlier at 4 ℃ overnight following the dilutions of: SMAD7 (1:500, SCBT, sc365846), CDK6 (1:500, SCBT, sc7961), ZEB1 (1:1000, CST, 70512S), STAT3 (1:500, SCBT, sc8019), TGIF2 (1:500, SCBT, sc81989), and GAPDH (1:2000, CST, 5174S), respectively. We washed the membrane three times for 10 min each in PBST and then incubated with horseradish peroxidase conjugated anti-rabbit or anti-mouse secondary antibody (1:5000) for 1 h at room temperature in a shaker. We further washed the membrane three times for 10 min each using PBST and used for imaging. The fluorescence intensity was measured using a commercial SuperSignal ELISA Femto Substrate (37075, IL, USA) in an IVIS-Lumina imaging system (Perkin Elmer, Bridgeville, PA, USA). We used the membranes processed using GAPDH antibody (Cell Signaling Technologies) as an internal control.

### *In vivo experiments in GBM mouse model*

**Hematoxylin and eosin staining.** At the final time point of *in vivo* experiments, we harvested the mice organs and fixed them in 4% paraformaldehyde overnight at 4 °C, and immersed them in 70% ethanol. We sent the samples to the Stanford Animal Histology Services for H&E staining, and we imaged them using a Nanozoomer (Hamamatsu, Japan).

**Table S1.** Significant miRNA expression changes observed in GBM versus normal brain tissue from GEO and TCGA-GBM databases.

| GBM cells | | | | | | | |
| --- | --- | --- | --- | --- | --- | --- | --- |
| miRNA ID | Downregulation  cancer versus normal (log_2_FC) | | | miRNA ID | Upregulation  cancer versus normal (log_2_FC) | | |
|  | GSE25631 | GSE90603 | TCGA |  | GSE25631 | GSE90603 | TCGA |
| miRNA-203 | -3.45 | -1.22 | -2.72 | miRNA-214 | 2.19 | 1.05 | 1.69 |
| miRNA-139 | -1.46 | -2.88 | -4.75 | miRNA-19b | 1.18 | 1.53 | 1.52 |
| miRNA-490 | -2.34 | -2.76 | -2.34 | miRNA-21 | 2.76 | 2.3 | 4.4 |
| miRNA-138 | -3.5 | -2.99 | -3 | miRNA-196a | 5.59 | 1.23 | 8.8 |
| miRNA-7 | -1.5 | -3.2 | -3.51 | miRNA-339 | 1.34 | 2.32 | 2.77 |
| miRNA-485 | -2.02 | -1.87 | -3.01 | miRNA-199b | 1.98 | 2.53 | 2.22 |
| miRNA-433 | -2.39 | -2.36 | -3.55 | miRNA-155 | 2.39 | 3.59 | 2.52 |
| miRNA-628 | -1.44 | -1.49 | -2.29 | miRNA-542 | 1.3 | 2.03 | 1.42 |
| miRNA-383 | -3.31 | -3.62 | -2.4 | miRNA-193a | 1.48 | 1.12 | 1.72 |
| miRNA-218 | -2.31 | -2.9 | -4.4 | miRNA-19a | 2.15 | 1.19 | 1.28 |
|  |  |  |  | miRNA-16-1 | 1.67 | 1.7 | 1.28 |
|  |  |  |  | miRNA-196b | 2.29 | 1.02 | 7.29 |
|  |  |  |  | miRNA-550* | 1.87 | 1.69 | 1.5 |
|  |  |  |  | miRNA-27a | 1.46 | 1.26 | 2.29 |

**Table S2.** Dysregulated miRNAs and their target genes.

| miRNA ID | Average log_2_FC | Therapeutic target genes from Pubmed | Therapeutic target genes from TargetScan and miRTarBase | Therapeutic pathways |
| --- | --- | --- | --- | --- |
| miR-203 | -2.46 | CD133, IFN, FGFR1, ZEB1, ADAMDEC1, SNAI2, ROBO1 | FGFR1OP, ZEB1, SNAI2, ROBO1 | Apoptosis, stemness, EMT, Cytokine signaling |
| miR-139-5p | -3.03 | ELTD1, ZEB1, ZEB2, Notch1, EIF4G2 | ZEB1, ZEB2, Notch1, EIF4G2 | Proliferation and apoptosis, invasion and metastasis |
| miR-490 | -2.48 | PRC2, EZH2, CHRM2, MMP9, CCL5, PIK3R1, ICAM1, ADAM17 and NOTCH1, TGFBR1 and TGIF2 | TGFBR1 | Proliferation, Invasion and metastasis, cytokine signaling |
| miR-138 | -3.16 | circFOXO3, NFAT5, EZH2, CDK6, E2F2 and E2F3 | NFAT5, EZH2, CDK6, E2F2, E2F3 | tumorigenesis |
| miR-218 | -2.605 | BMI1, TET2, HIF2a, EGFR, PIK3CA, ARAF, STAT3, BCLAF1, LEF1 | Bmi1, ARAF | stemness, RTK, mesenchymal, growth and progression, invasiveness |
| miR-7 | -2.73 | YY1, TFF3, DR5, TRAIL | RYBP | stemness, migration, apoptosis |
| miR-485-5p | -2.3 | TPD52L2, TP53, PAK4 | TPD52L2, PAK4 | tumorigenesis |
| miR-433 | -2.76 | cAMp, KRAS, MAPk4, p21 activated kinase | CREB1, KRAS, PAK4 |  |
| miR-628 | -1.74 | circPCMTD1, HMGB3 | circPCMTD1, HMGB3 | proliferation, migration, EMT |
| miR-383 | -3.11 | Nothing was found | - | - |
| miR-214-5p | 1.64 | PPARa, E2F2, CXCR5 | E2F2, CXCR5 | Proliferation, invasion and metastasis |
| miR-19b | 1.41 | PTEN | PTEN | Apoptosis, stemness |
| miR-21-3p | 3.15 | MPS1 -> PDCD4, MSH2, SMAD3 TGF-b/SMAd, IGFBP3, PTEN | PDCD4, MSH2, TGF-b2 | Proliferation, tumorigenesis, Cytokine singling |
| miR-196a-5p | 5.20 | ZMYND11, IKBa NFkB | ZMYND11 | Proliferation, apoptosis, cytokine signaling |
| miR-27a | 1.36 | FOXO3a, BTG2 | FOXO3, BTG2 | Proliferation and invasion |
| miR-339-5p | 2.14 | TUSC3 | - | - |
| miR-199b-5p | 2.24 | Hes-1 | - | - |
| miR-155 | 2.83 | FAM133A | - | invasion |
| miR-542 | 1.58 | PTPN1 | - | carcinogenesis, invasion |
| miR-193a-3p | 1.44 | Cyclin D1 | - | cell cycle progression |
| miR-19a | 1.54 | TET2, PPARa | PPARa | proliferation, metastasis |
| miR-16-1-3p | 1.55 | Nothing was found | - | - |
| miR-196b-5p | 3.53 | Nothing was found | - | - |
| miR-550* | 1.68 | Nothing was found | - | - |

Four of the miRNAs in this table have an adj p-value >0.05 in at least one of the datasets: miR-214, miR-196a, miR-218, miR-27a.

**Table S3.** The list of miRNAs selected for therapeutic applications in GBM and their therapeutic target genes.

| **miRNA ID** | **Average log_2_FC** | **Therapeutic target genes** | **Therapeutic pathways** |
| --- | --- | --- | --- |
| miRNA-138 | -3.16 | CDK6, EZH2, CCND1, E2F2 | Proliferation and apoptosis |
| miRNA-139 | -3.03 | NOTCH1, EIF4G2, ZEB1, MCL1 | Proliferation and apoptosis, Invasion and metastasis, Stemness |
| miRNA-203 | -2.46 | FGFR1, SNAI2, ZEB1 | Stemness |
| miRNA-218 | -2.60 | ROBO1, IKKB, STAT3, BMI1 | Proliferation and apoptosis, Cytokine signaling, Invasion, Stemness |
| miRNA-490 | -2.48 | CCND1, TGIF2, NOTCH1, ADAM17 | Proliferation and apoptosis, Stemness |
| miRNA-21 | 3.15 | RASGRP1, SMAD7 | Cytokine signaling, Invasion and metastasis |

**Table S4.** Survival analysis of the dysregulated miRNAs from OncoLnc.

| miRNA ID | Average log_2_FC | Cox Coefficient | FDR corrected  p-value | Logrank  p-value (33-33) |
| --- | --- | --- | --- | --- |
| miR-203 | -2.46 | 0.634 | 8.57e-1 | 0.0716 |
| miR-139-5p | -3.03 | 0.072 | 9.19e-1 | 0.694 |
| miR-490 | -2.48 | -0.167 | 9.62e-1 | 0.125 |
| miR-138 | -3.16 | 0.038 | 9.62e-1 | 0.287 |
| miR-7 | -2.73 | 0.068 | 8.57e-1 | 0.193 |
| miR-485-5p | -2.3 | 0.484 | 8.73e-1 | 0.942 |
| miR-433 | -2.76 | -0.011 | 9.96e-1 | 0.192 |
| miR-628 | -1.74 | 0.32 | 8.73e-1 | 0.403 |
| miR-383 | -3.11 | 0.42 | 2.45e-1 | 0.209 |
| miR-218 | -2.605 | 0.174 | 4.27e-1 | 0.469 |
| miR-214-5p | 1.64 | -0.021 | 9.72e-1 | 0.719 |
| miR-19b | 1.41 | -0.064 | 9.19e-1 | 0.00682 |
| miR-21-3p | 3.15 | 0.056 | 9.07e-1 | 0.0491 |
| miR-196a-5p | 5.20 | 0.196 | 1.48e-1 | 0.0156 |
| miR-339-5p | 2.14 | 0.028 | 9.78e-1 | 0.181 |
| miR-199b-5p | 2.24 | 0.067 | 9.19e-1 | 0.766 |
| miR-155 | 2.83 | 0.114 | 7.50e-1 | 0.0129 |
| miR-542 | 1.58 | 0.183 | 8.57e-1 | 0.325 |
| miR-193a-3p | 1.44 | 0.061 | 9.19e-1 | 0.0763 |
| miR-19a | 1.54 | -0.056 | 9.19e-1 | 0.000566 |
| miR-16-1-3p | 1.55 | 0.046 | 9.62e-1 | 0.581 |
| miR-196b-5p | 3.53 | 0.076 | 9.19e-1 | 0.498 |
| miR-550* | 1.68 | 0.206 | 1.80e-1 | Not found |
| miR-27a | 1.36 | -0.063 | 9.19e-1 | 0.9 |

**Table S5.** Univariate and multivariate cox regression analysis of the survival-related genes. HR: Hazard ratio, se: standard error, coef = coefficient.

| Genes | Univariate analysis | | Multivariate analysis | |
| --- | --- | --- | --- | --- |
|  | HR (se(coef)) | p-value | HR (se(coef)) | p-value |
| CDK6 | -1.81e-5 (4.27e-5) | 0.67 | -1.85e-5 (3.77e-5) | 0.62 |
| ZEB1 | -1.95e-4 (8.02e-5) | 0.014 | -1.99e-4 (8.4e-5) | 0.017 |
| FGFR1 | 1.32e-4 (4.80e-5) | 0.005 | 1.21e-4 (5.84e-5) | 0.038 |
| STAT3 | 1.81e-4 (9.22e-5) | 0.048 | 4.85e-5 (1.08e-4) | 0.65 |
| TGIF2 | -3.08e-4 (3.12e-4) | 0.32 | -4.4e-4 (3.73e-4) | 0.23 |
| SMAD7 | -5.0e-4 (6.12e-4) | 0.41 | -2.63e-4 (7.24e-4) | 0.71 |

**Table S6.** Different combinations of miRNAs selected for evaluation of GBM treatment.

| Condition | miR-138 | miR-139 | miR-203 | miR-218 | miR-490 | AmiR-21 | Control NP |
| --- | --- | --- | --- | --- | --- | --- | --- |
| 1 | - | - | - | + | - | - | + |
| 2 | - | - | - | + | - | + | + |
| 3 | - | - | + | + | - | - | + |
| 4 | - | - | - | + | + | - | + |
| 5 | - | - | - | + | + | + | + |
| 6 | + | - | - | + | - | + | + |
| 7 | - | + | - | + | + | - | + |
| 8 | - | + | - | + | + | + | + |
| 9 | + | + | - | + | + | - | + |
| 10 | + | + | - | + | + | + | + |
| 11 | - | - | - | - | - | - | + |
| 12 | - | - | - | - | - | - | - |

**Table S7.** Cell cycle analysis for selected conditions in miRNA GBM study for different doses of TMZ. Figure S3 contains the graphs representing different cell cycle phases.


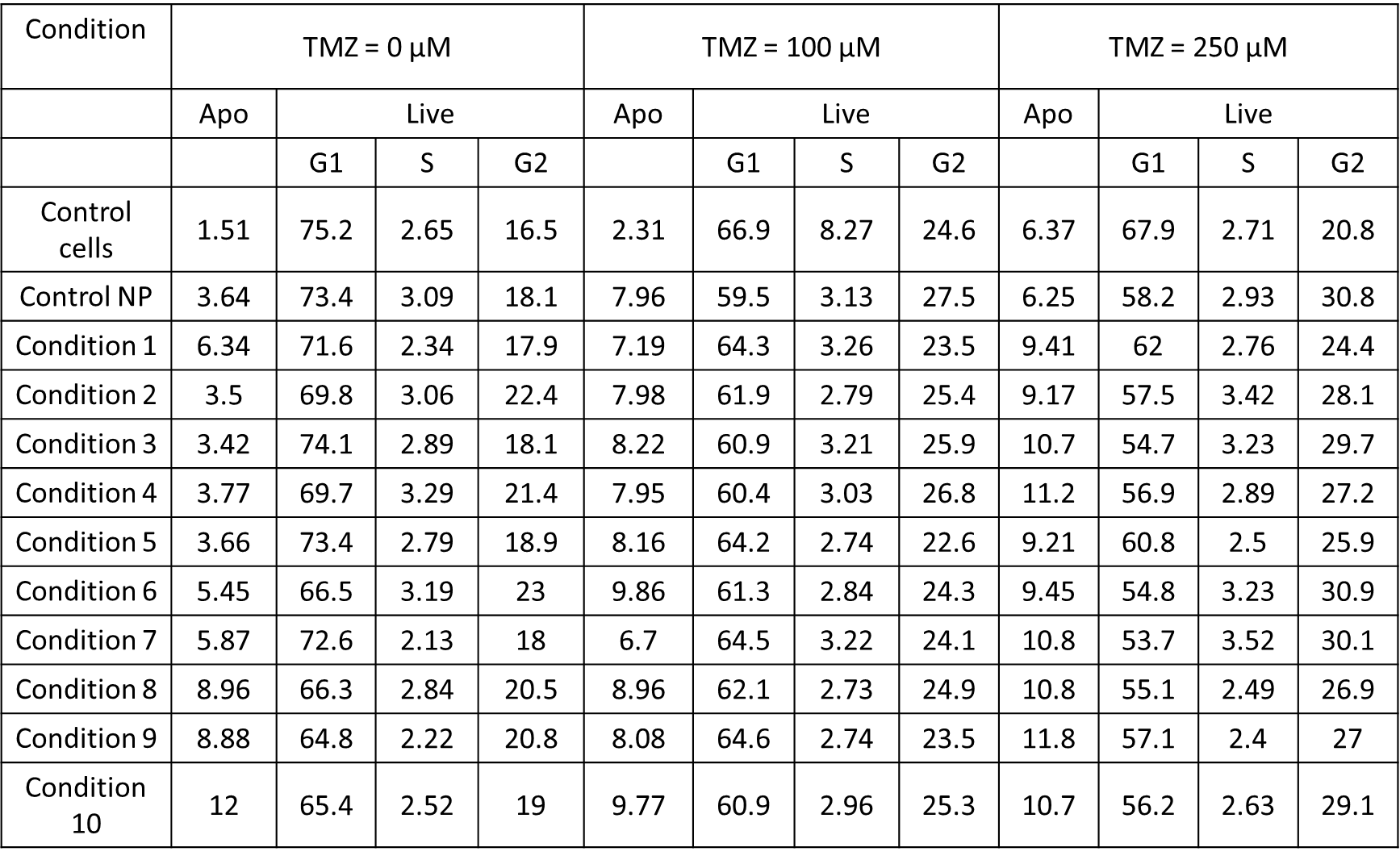


**Table S8.** Cell cycle analysis for selected conditions in miRNA GBM study after pretreatment with different combinations of miRNAs and treatment with two consecutive doses of TMZ. Figure S4 contains the graphs representing different cell cycle phases.


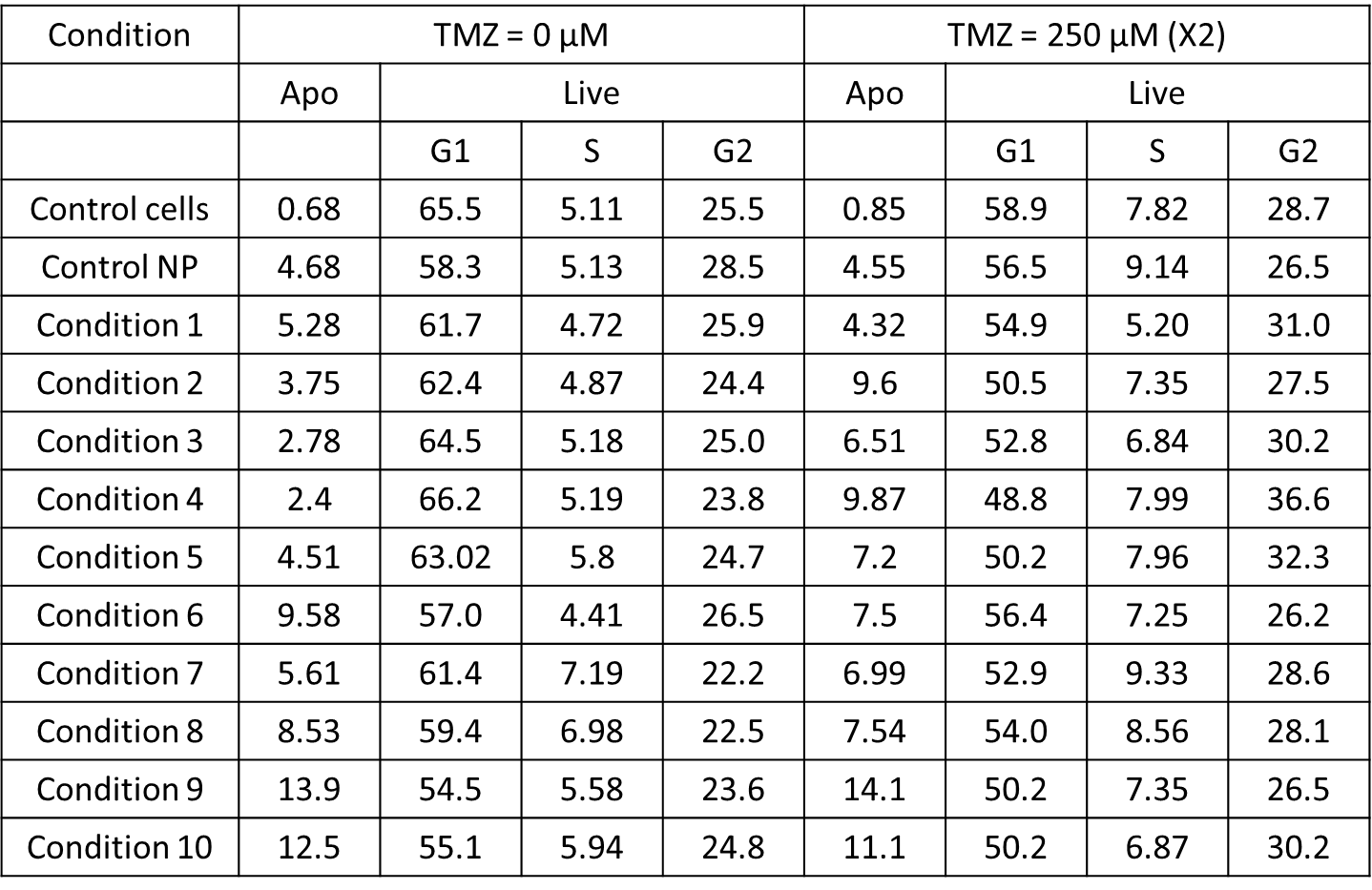


**Table S9.** Cell cycle analysis for selected conditions in miRNA GBM study after pretreatment with different combinations of miRNAs and treatment with doxorubicin. Figure S5 contains the graphs which represent different cell cycle phases.


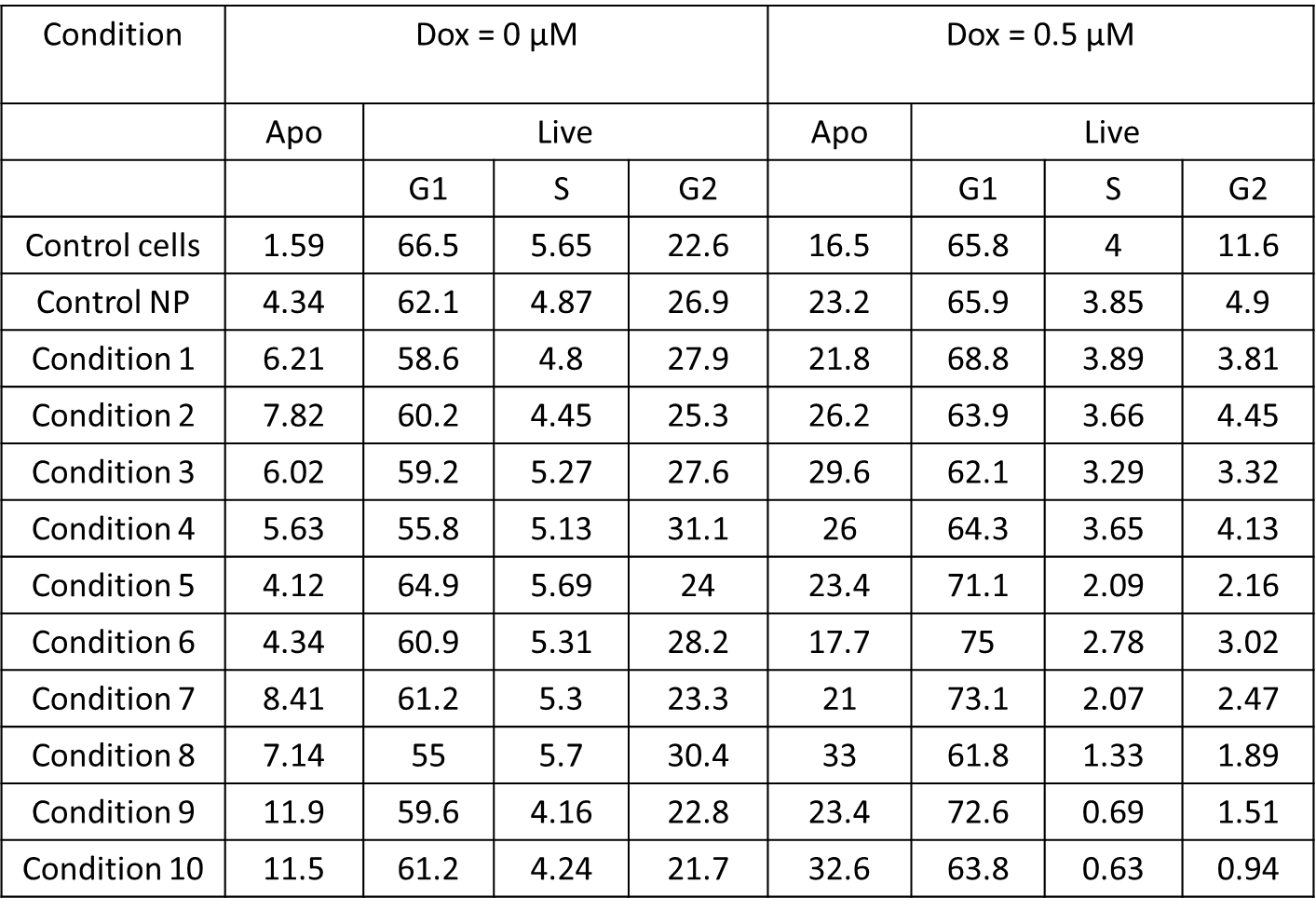


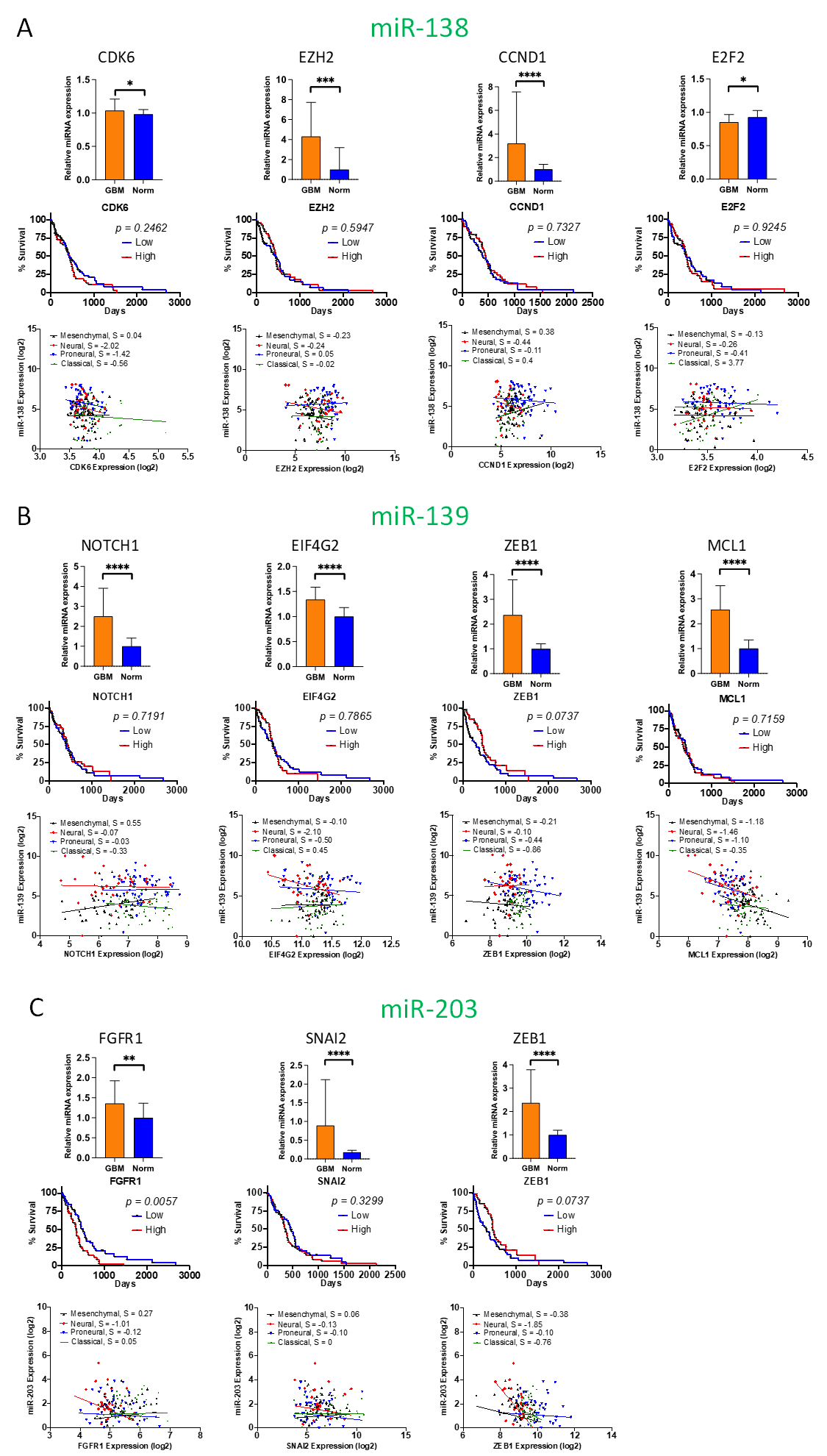


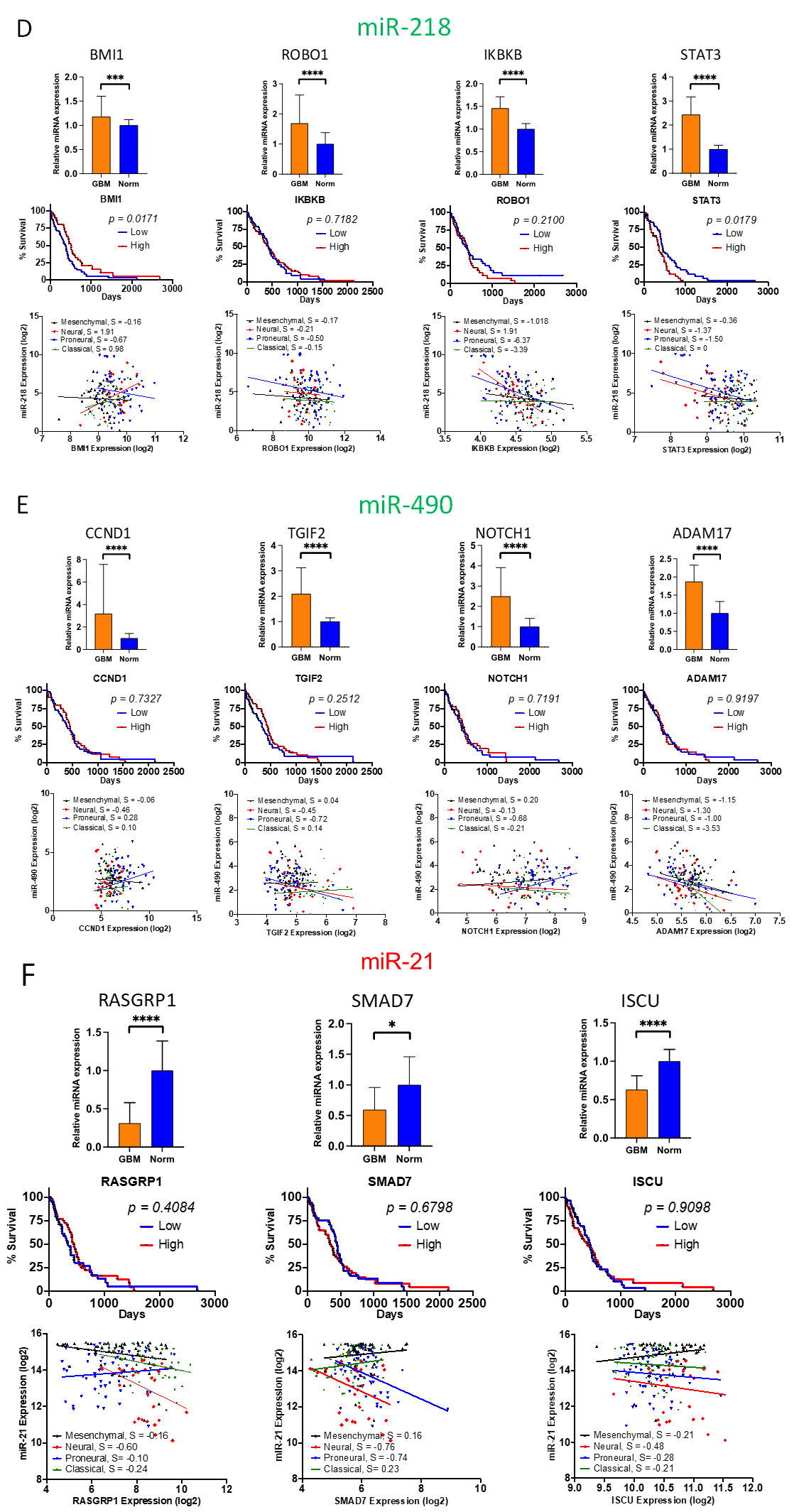


**Figure S1.** **Survival curves of the therapeutic target genes for each of the six selected miRNAs, and correlation of each therapeutic target genes with corresponding miRNA expression.** Survival curves and expression data are derived from **the** TCGA_GBM dataset. Correlation between the target gene and miRNA expressions were studied in four different GBM subtypes: mesenchymal, neural, proneural, and classical. **(A)** miR-138. **(B)** miR-139. **(C)** miR-203. **(D)** miR-218. **(E)** miR-490. **(F)** miR-21. In survival analysis plots, *p* indicates the p-value. Low and High refer to top and bottom 33% of expressions. In correlation plots, S indicates the slope of the regression line fitted on the expression data. A negative slope is expected if the gene is targeted by the miRNA.


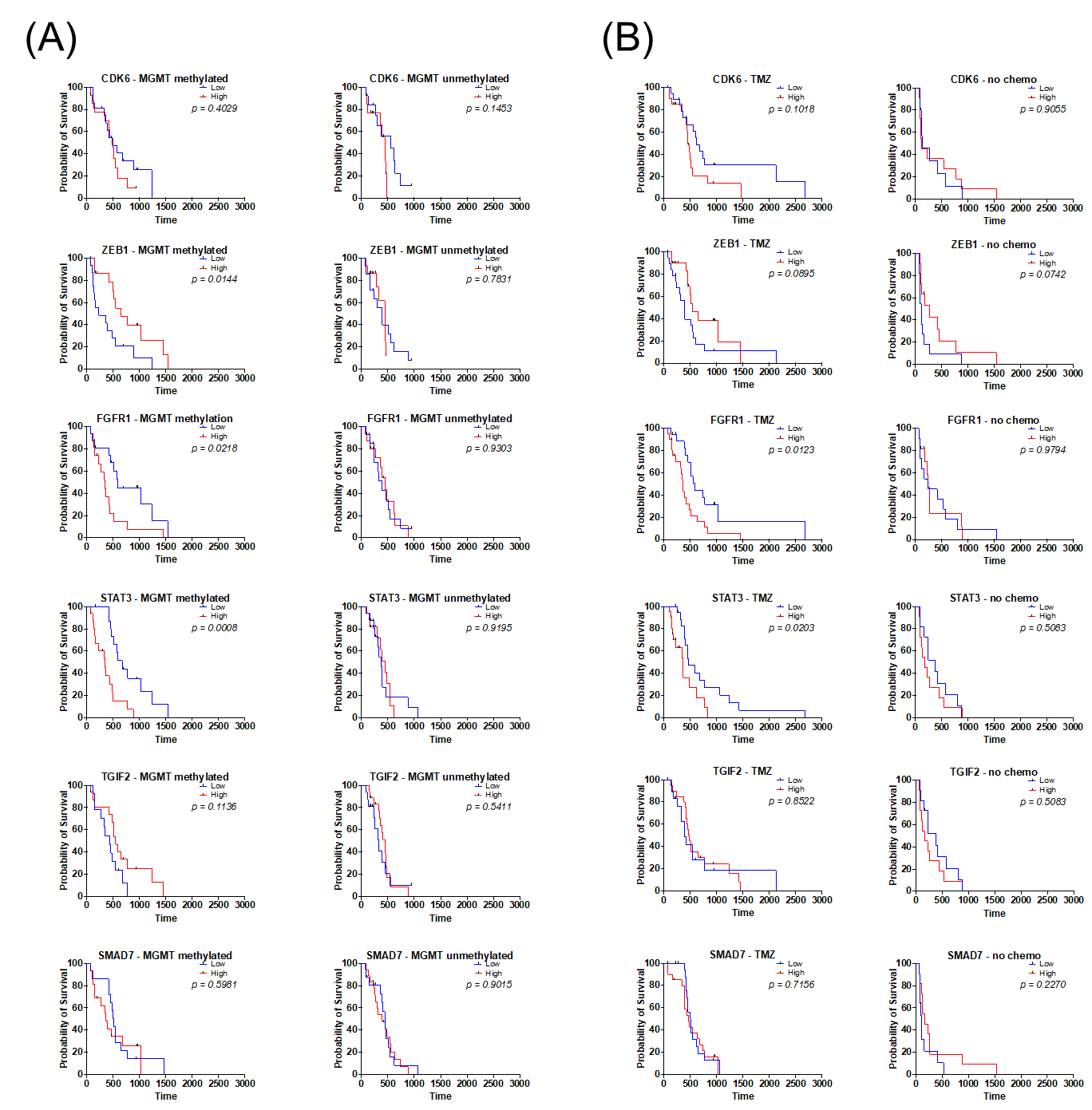


**Figure S2.** **Survival curves of the therapeutic target genes for each of the six selected miRNAs. (A) MGMT promoter methylated versus unmethylated groups and (B) TMZ treated versus non chemo- treated groups.** Survival curves and expression data are derived from the TCGA_GBM dataset. In survival analysis plots, *p* indicates the p-value. Low and High refer to top and bottom 20% of expressions.


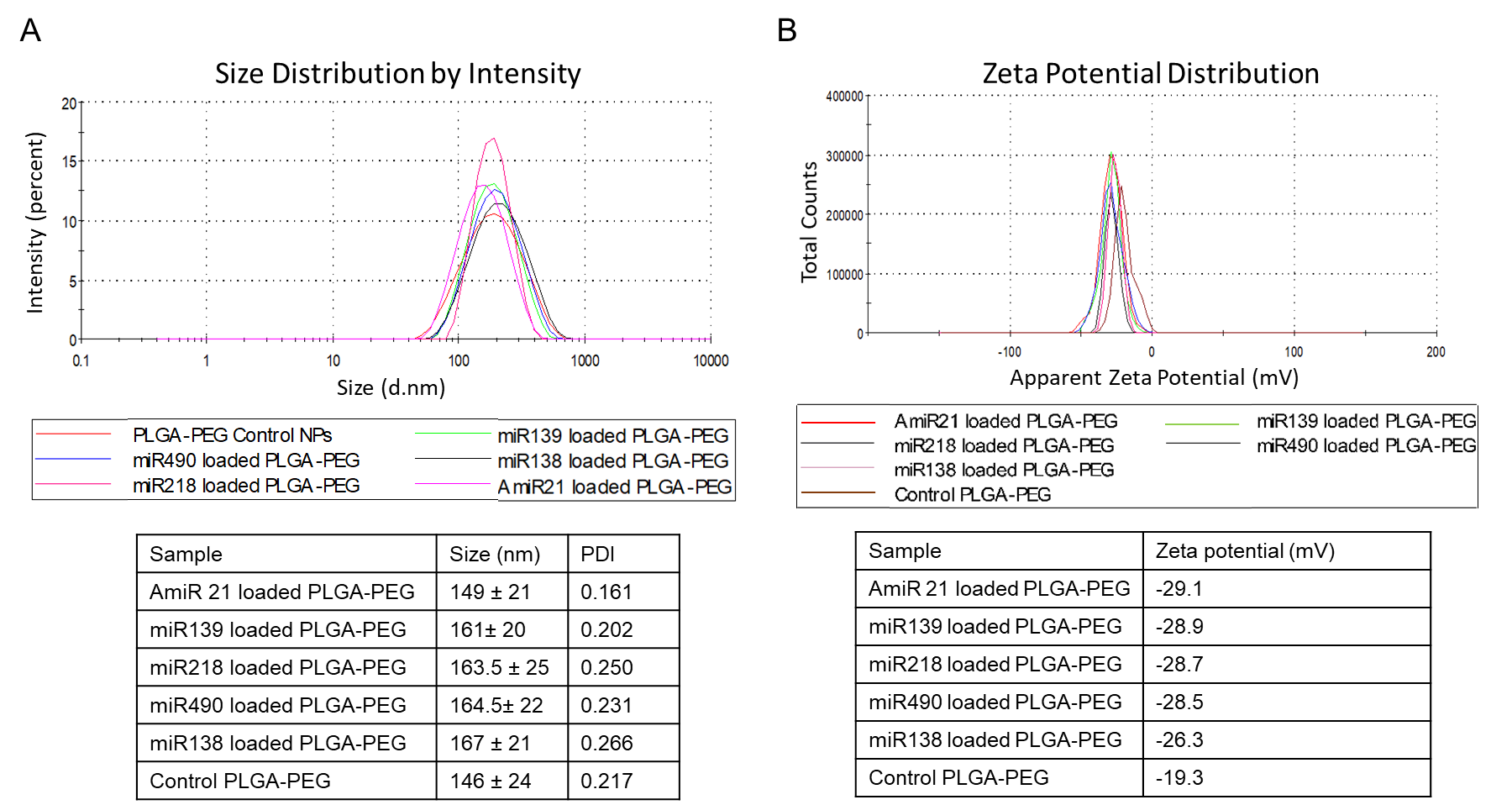


**Figure S3. Characterization of miRNA loaded PLGA-PEG NPs.** **(A)** Size distribution showed a narrow size range of the NPs. **(B)** Zeta potential distribution showed negative zeta potential for all the NPs.


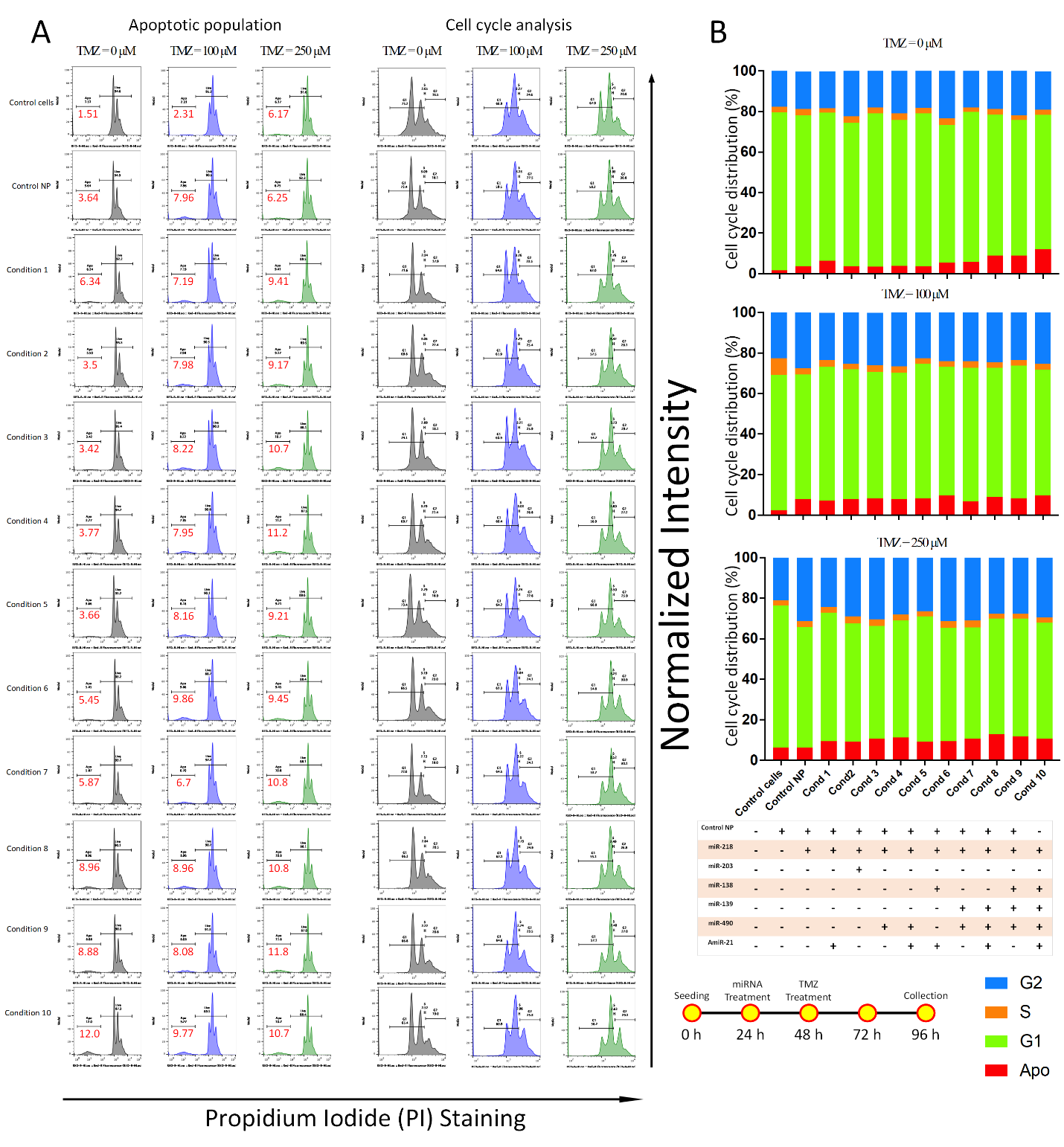


**Figure S4.** FACS data analysis on U87-MG cell line to find the apoptotic and live populations after pretreatment with miRNAs and subsequent treatment with different doses of TMZ. Cells were collected 48 h after incubation with drug.


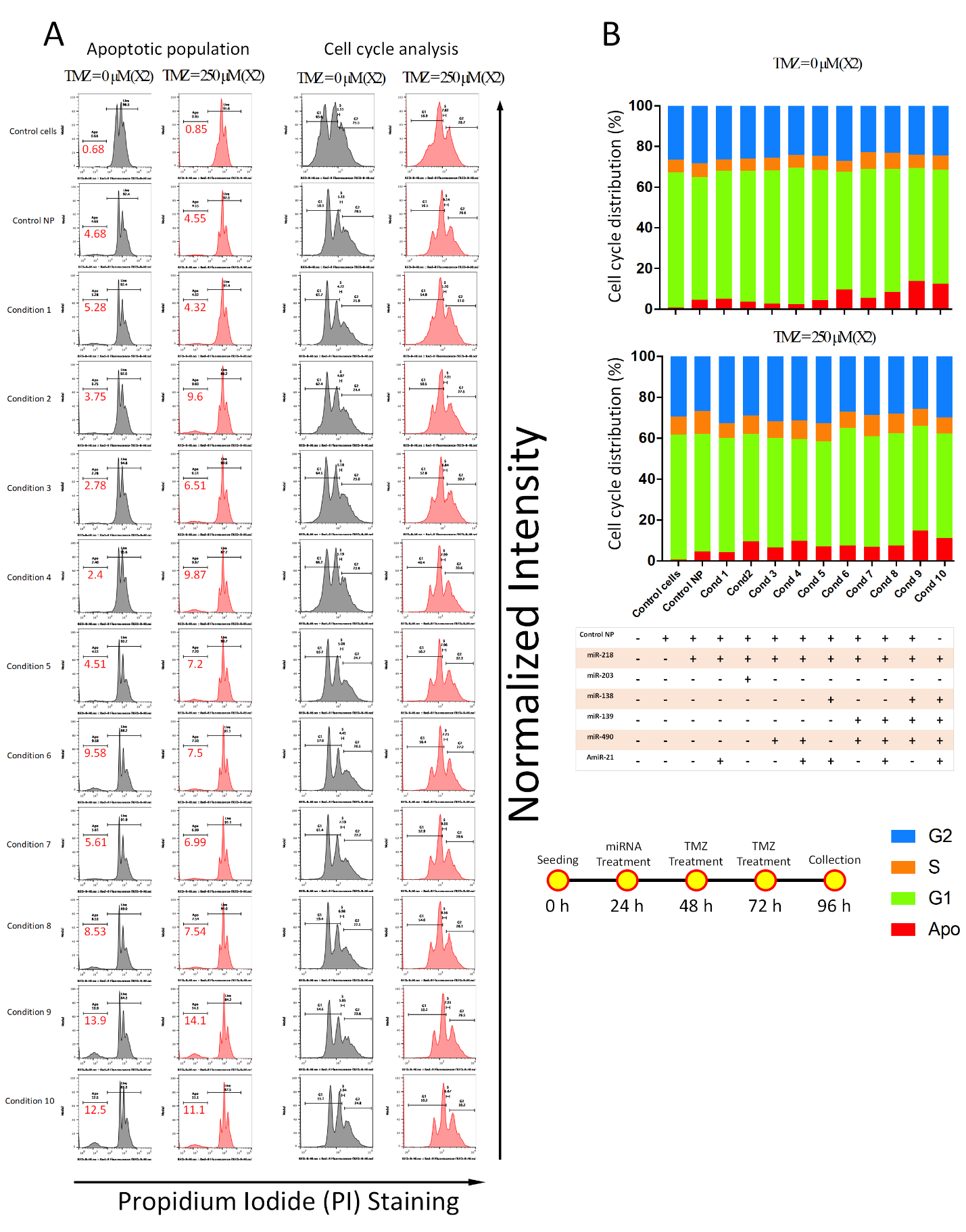


**Figure S5.** FACS data analysis on U87-MG cell line to find the apoptotic and live populations after pretreatment with miRNAs and subsequent treatment with two consecutive doses of 250 μM TMZ. Cells were collected 24 h after incubation with the second dose.


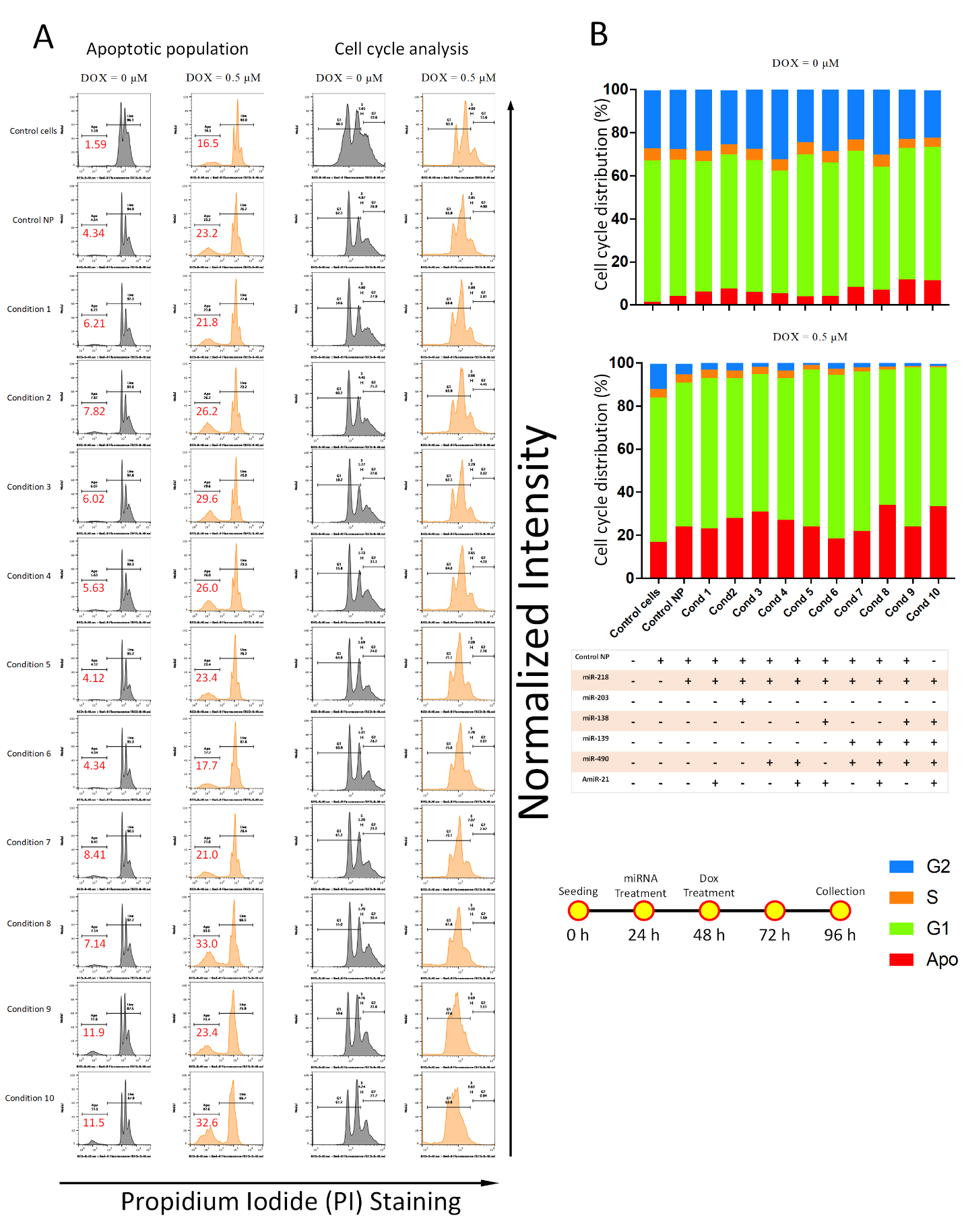


**Figure S6.** FACS data analysis on U87-MG cell line to find the apoptotic and live populations after pretreatment with miRNAs and subsequent treatment with 0.5 μM DOX. Cells were collected 48 h after incubation with drug.


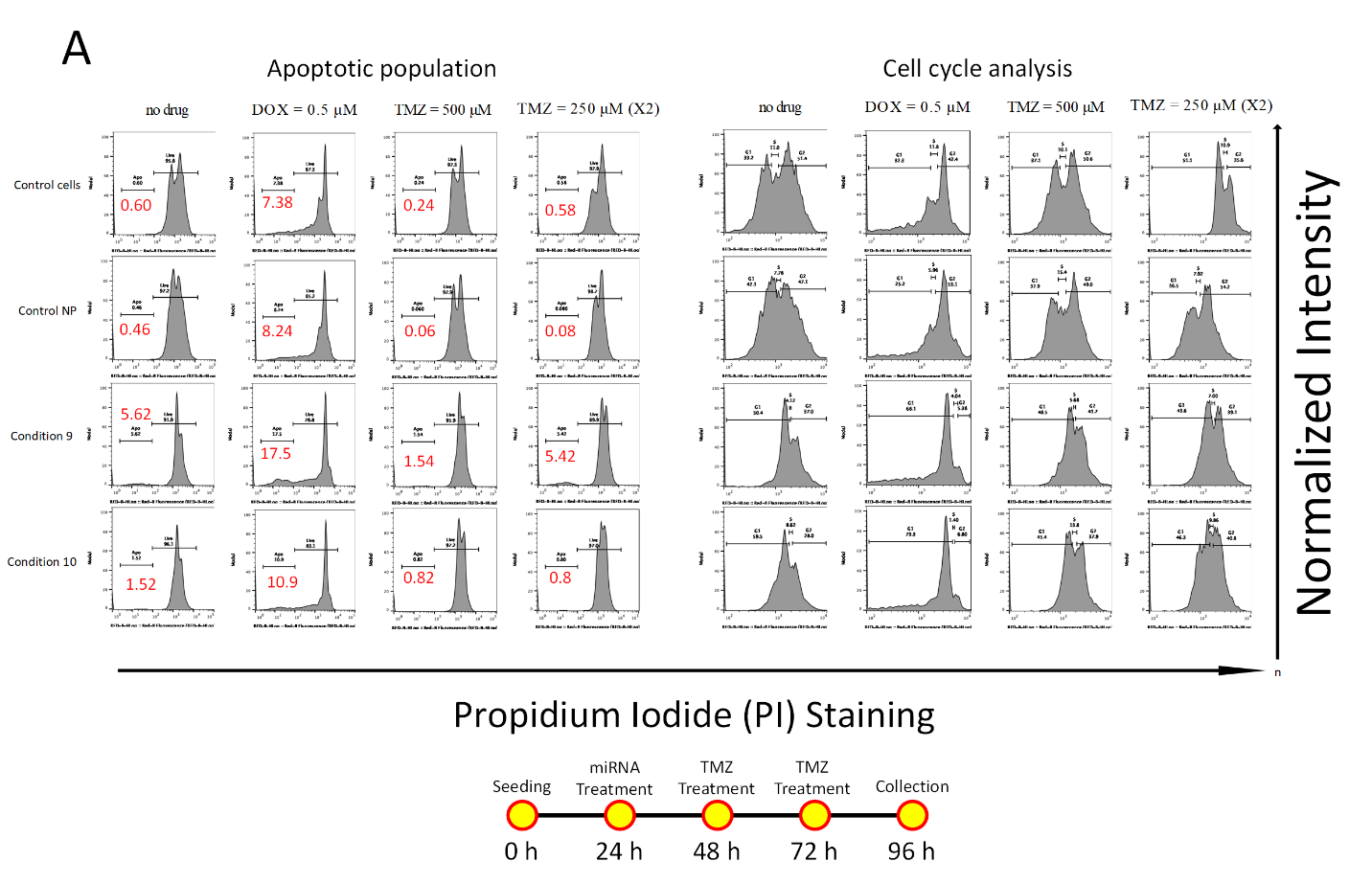


**Figure S7.** FACS data analysis on T98g cell line to find the apoptotic and live populations after pretreatment with miRNAs and subsequent treatment with TMZ or DOX.


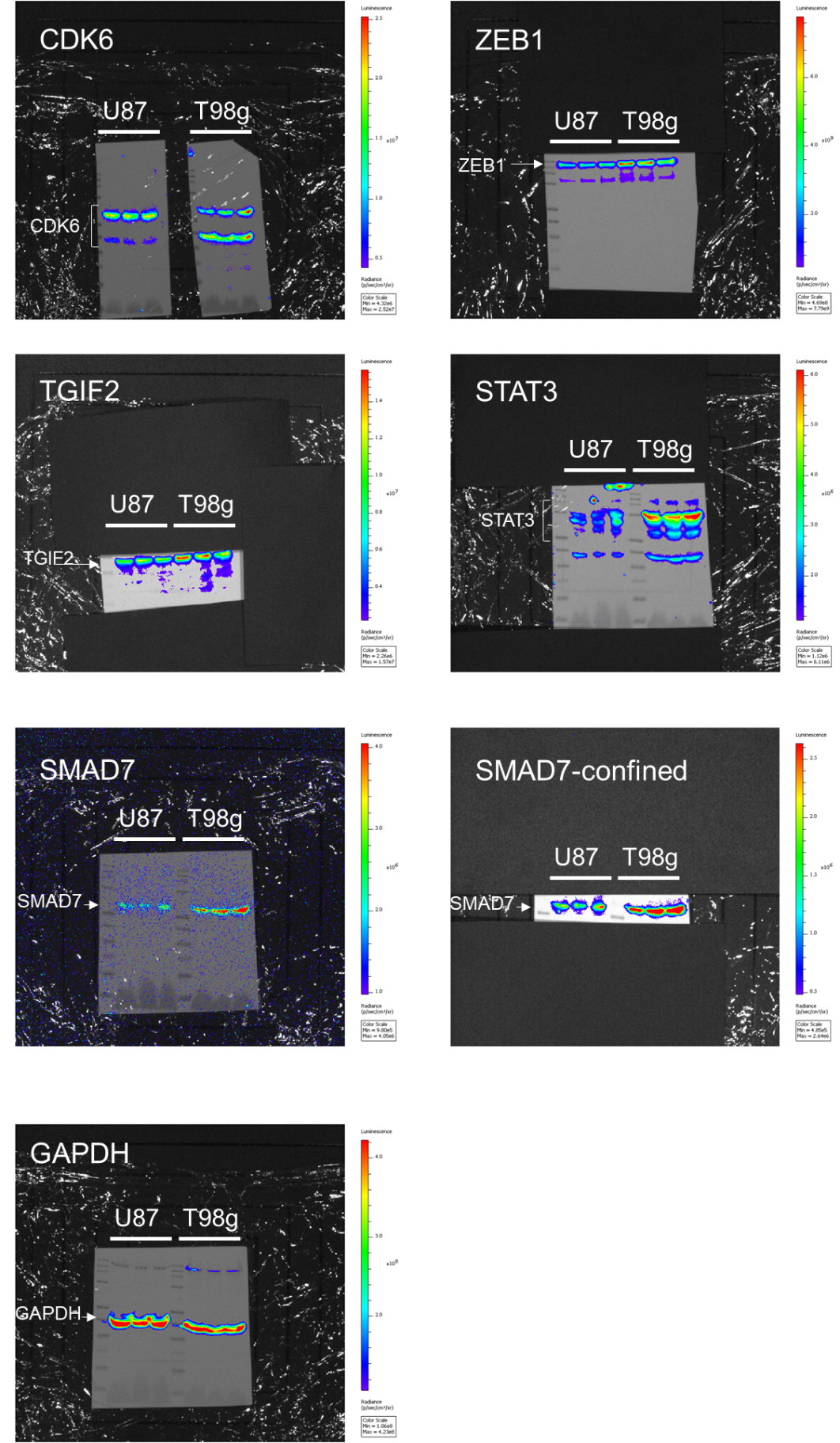


**Figure S8. Supportive information for Fig. 4B in the main article.** Whole western blotting membranes to show the location of each band and the intensities.


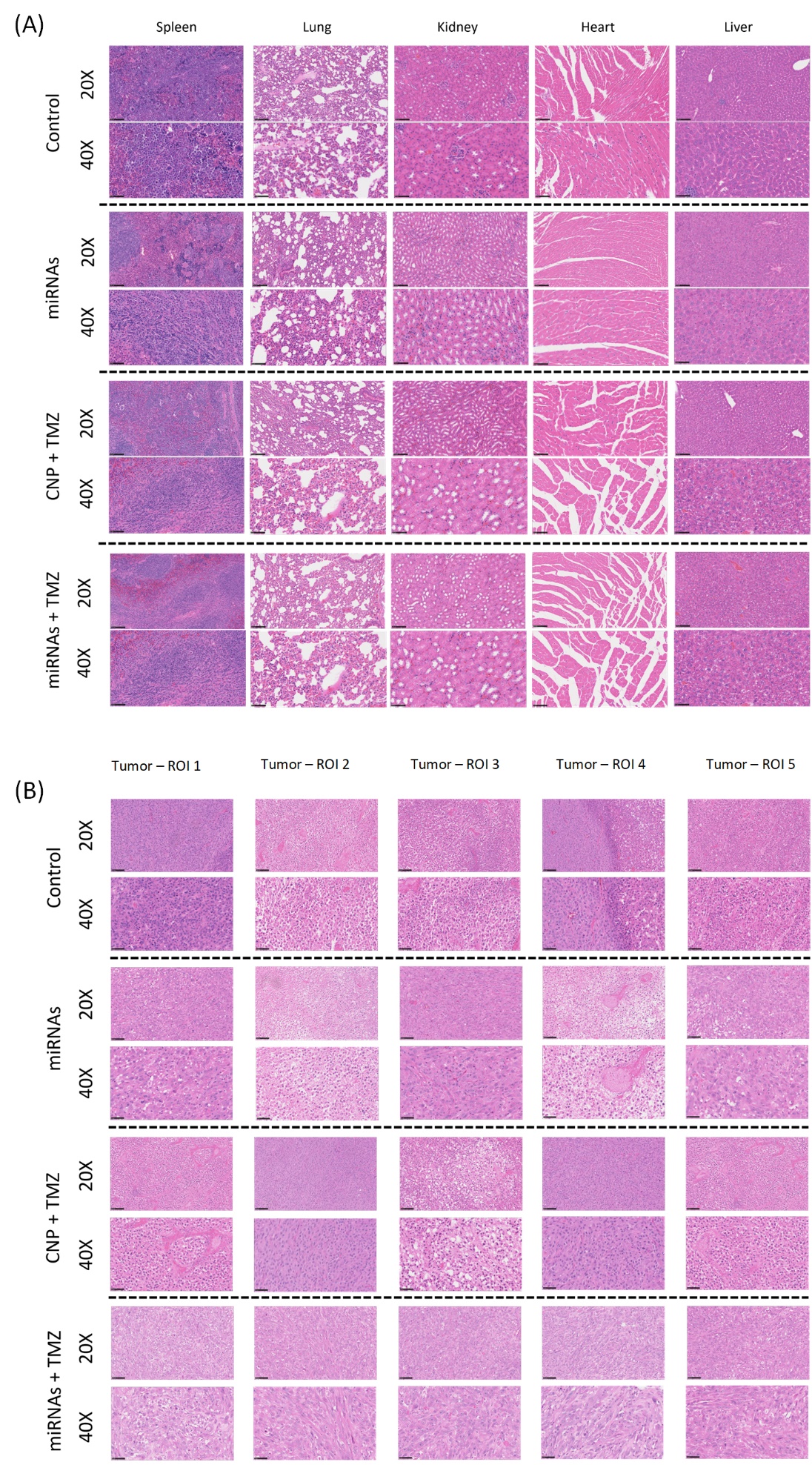


**Figure S9. Hematoxylin and eosin (H&E) stain of the tissues.** **(A)** H&E stained histologic sections of major organs in mice in different treatment groups. There was no tissue damage indicating the absence of toxicity. **(B)** H&E stained histologic sections of 5 different region of interest from tumors in mice in different treatment groups. Control mice did not receive any treatment, miRNAs received combination #10 including (antimiRNA-21, miRNA-138, miRNA-139, miRNA-218, and miRNA-490), the CNP plus TMZ group received control NPs and TMZ, the miRNAs plus TMZ group received condition #10 and TMZ.


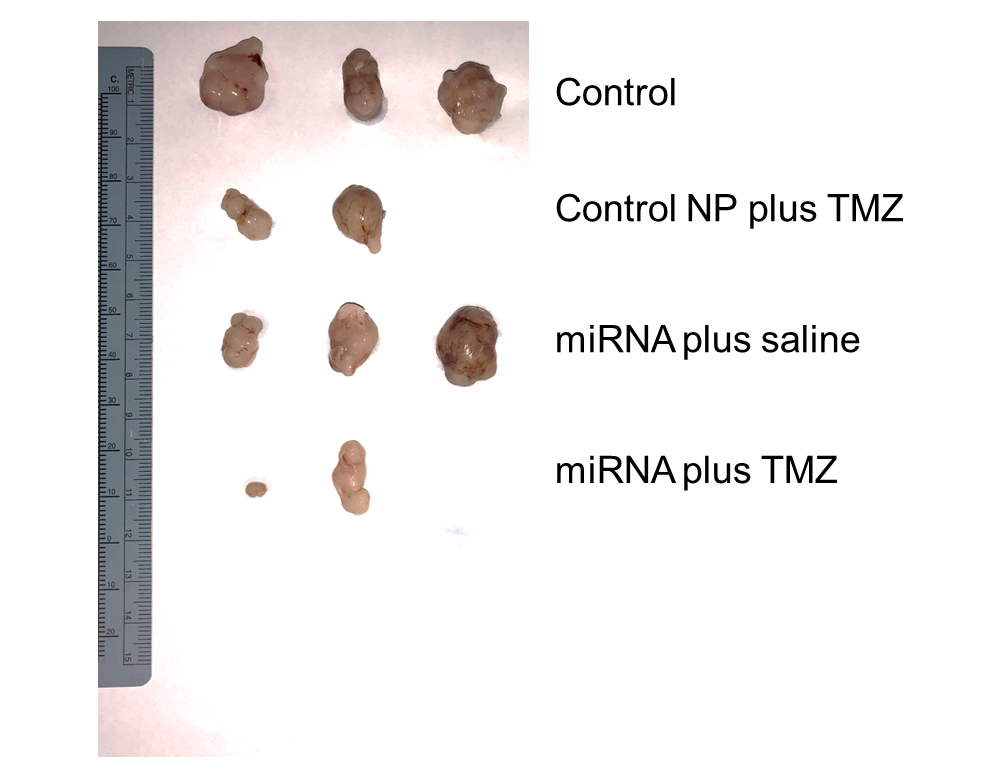


**Figure S10. Representative tumors in each treatment group.**
